# Association of Convalescent Plasma Therapy With Survival in Patients With Hematologic Cancers and COVID-19

**DOI:** 10.1001/jamaoncol.2021.1799

**Published:** 2021-06-17

**Authors:** Michael A. Thompson, Jeffrey P. Henderson, Pankil K. Shah, Samuel M. Rubinstein, Michael J. Joyner, Toni K. Choueiri, Daniel B. Flora, Elizabeth A. Griffiths, Anthony P. Gulati, Clara Hwang, Vadim S. Koshkin, Esperanza B. Papadopoulos, Elizabeth V. Robilotti, Christopher T. Su, Elizabeth M. Wulff-Burchfield, Zhuoer Xie, Peter Paul Yu, Sanjay Mishra, Jonathon W. Senefeld, Dimpy P. Shah, Jeremy L. Warner

**Affiliations:** 1 Department of Medicine, Aurora Cancer Care, Advocate Aurora Health, Milwaukee, Wisconsin; 2 Department of Medicine, Washington University School of Medicine in St Louis, St Louis, Missouri; 3 Department of Urology, Mays Cancer Center at UT Health San Antonio MD Anderson, San Antonio, Texas; 4 Department of Medicine, University of North Carolina Lineberger Comprehensive Cancer Center, Chapel Hill; 5 Department of Anesthesiology and Perioperative Medicine, Mayo Clinic, Rochester, Minnesota; 6 Department of Medicine, Dana-Farber Cancer Institute, Boston, Massachusetts; 7 Oncology Research Program, St Elizabeth Healthcare, Edgewood, Kentucky; 8 Department of Medicine, Roswell Park Comprehensive Cancer Center, Buffalo, New York; 9 Department of Medicine, Stamford Hospital, Stamford, Connecticut; 10 Department of Internal Medicine, Henry Ford Cancer Institute, Detroit, Michigan; 11 Department of Medicine, UCSF Helen Diller Family Comprehensive Cancer Center, University of California, San Francisco; 12 Department of Medicine, Memorial Sloan Kettering Cancer Center, New York, New York; 13 Department of Internal Medicine, University of Michigan Rogel Cancer Center, Ann Arbor; 14 Department of Internal Medicine, University of Kansas Medical Center, Kansas City; 15 Mayo Clinic Cancer Center, Rochester, Minnesota; 16 Hartford Health Care, Farmington, Connecticut; 17 Vanderbilt-Ingram Cancer Center, Nashville, Tennessee; 18 Department of Population Health Sciences, Mays Cancer Center at UT Health San Antonio MD Anderson, San Antonio, Texas; 19 Departments of Medicine and Biomedical Informatics, Vanderbilt University Medical Center, Nashville, Tennessee

## Abstract

**Importance:**

COVID-19 is a life-threatening illness for many patients. Prior studies have established hematologic cancers as a risk factor associated with particularly poor outcomes from COVID-19. To our knowledge, no studies have established a beneficial role for anti–COVID-19 interventions in this at-risk population. Convalescent plasma therapy may benefit immunocompromised individuals with COVID-19, including those with hematologic cancers.

**Objective:**

To evaluate the association of convalescent plasma treatment with 30-day mortality in hospitalized adults with hematologic cancers and COVID-19 from a multi-institutional cohort.

**Design, Setting, and Participants:**

This retrospective cohort study using data from the COVID-19 and Cancer Consortium registry with propensity score matching evaluated patients with hematologic cancers who were hospitalized for COVID-19. Data were collected between March 17, 2020, and January 21, 2021.

**Exposures:**

Convalescent plasma treatment at any time during hospitalization.

**Main Outcomes and Measures:**

The main outcome was 30-day all-cause mortality. Cox proportional hazards regression analysis with adjustment for potential confounders was performed. Hazard ratios (HRs) are reported with 95% CIs. Secondary subgroup analyses were conducted on patients with severe COVID-19 who required mechanical ventilatory support and/or intensive care unit admission.

**Results:**

A total of 966 individuals (mean [SD] age, 65 [15] years; 539 [55.8%] male) were evaluated in this study; 143 convalescent plasma recipients were compared with 823 untreated control patients. After adjustment for potential confounding factors, convalescent plasma treatment was associated with improved 30-day mortality (HR, 0.60; 95% CI, 0.37-0.97). This association remained significant after propensity score matching (HR, 0.52; 95% CI, 0.29-0.92). Among the 338 patients admitted to the intensive care unit, mortality was significantly lower in convalescent plasma recipients compared with nonrecipients (HR for propensity score–matched comparison, 0.40; 95% CI, 0.20-0.80). Among the 227 patients who required mechanical ventilatory support, mortality was significantly lower in convalescent plasma recipients compared with nonrecipients (HR for propensity score–matched comparison, 0.32; 95% CI, 0.14-0.72).

**Conclusions and Relevance:**

The findings of this cohort study suggest a potential survival benefit in the administration of convalescent plasma to patients with hematologic cancers and COVID-19.

## Key Points

**Question:**

Is convalescent plasma therapy associated with improved outcomes of hospitalized patients with COVID-19 and hematologic cancer?

**Findings:**

In this cohort study of 966 patients with hematologic cancer and COVID-19, after adjustment for potential confounding factors, convalescent plasma treatment was associated with a significantly improved 30-day mortality in the 143 individuals who received it. This association remained significant after propensity score matching.

**Meaning:**

These findings suggest a potential survival benefit in the administration of convalescent plasma to patients with hematologic cancers and COVID-19.

## Teaser

This cohort study evaluated the association of convalescent plasma treatment with 30-day mortality in hospitalized adults with hematologic cancers and COVID-19.

## Introduction

Since initial reports in late 2019, SARS-CoV-2 has infected more than 100 million people worldwide and caused more than 2 million deaths by early 2021.^1^ To date, data guiding COVID-19 therapies have largely arisen from large-scale studies^2,3^ of healthy adults. Patients with hematologic cancers represent a distinctive subset of patients with COVID-19 caused by immune deficits associated with both the diseases themselves and their treatments. Hematologic cancers have been consistently associated with increased COVID-19 mortality and other complications.^4,5,6^

Antibody-based immunity is an important correlate of SARS-CoV-2 recovery and vaccine-associated prevention. Hematologic cancers are associated with defects in humoral and cellular immunity that may contribute to adverse COVID-19 outcomes. Impaired antibody function is a well-described complication of plasma cell neoplasms, chronic lymphocytic leukemia (CLL), and other lymphoid cancers. Treatment of hematologic cancers often exacerbates these immune defects; for example, rituximab targets the pan-B cell marker CD20 and is highly effective therapy for B-cell cancers. However, B-cell depletion can cause lymphopenia and hypogammaglobulinemia and is associated with more severe COVID-19.^7^ Lymphopenia is known to be associated with more severe COVID-19.^8^

Antibody therapy using COVID-19 convalescent plasma was associated with a therapeutic benefit in a general patient population^9^ and older patients^10^ when high titer units were administered early in the disease. A negative prospective randomized trial included only 4 patients with hematologic cancers in the convalescent plasma group.^11^ In patients with immunodeficiency, case reports have noted exceptional improvements in clinical status after convalescent plasma therapy, even after relatively late infusion.^12^ Given the absence of definitive prospective trial data in patients with hematologic cancers, we conducted a retrospective cohort study to evaluate the hypothesis that convalescent plasma therapy can correct defects in humoral deficiency and improve outcomes.

## Methods

### Setting and Participants

The COVID-19 and Cancer Consortium (CCC19) is an international consortium aimed at understanding the clinical impact of COVID-19 in patients with cancer through a Vanderbilt University Institutional Review Board–exempted comprehensive registry. The methods for CCC19 have been described and published previously.^13^ We analyzed data from hospitalized US adults with a current or past diagnosis of hematologic cancers diagnosed with confirmed or suspected SARS-CoV-2 infection in 2020 and reported from March 17, 2020, to January 21, 2021 (full list of contributors is in the eAppendix in Supplement 1). Treatment exposure was defined as receiving convalescent plasma at any time during the COVID-19 illness. The exclusion criteria were incomplete follow-up resulting in unknown death status, unknown or missing convalescent plasma exposure, age younger than 18 years, mild COVID-19 not requiring hospitalization, and non-US residence. The following data elements were obtained: age, sex, race and ethnic groups, smoking status, comorbidities, the first recorded absolute lymphocyte count, type of hematologic cancer, cancer status at COVID-19 diagnosis, Eastern Cooperative Oncology Group (ECOG) performance status before COVID-19, receipt and timing of anticancer treatment, baseline COVID-19 severity, level of care required, other anti–COVID-19 therapies (ie, corticosteroids, remdesivir, tocilizumab, and hydroxychloroquine), and US Census region of patient's residence. Race and ethnic groups were as reported in the electronic health record of the patients and were included because of numerous reports of racial and ethnic disparities in patients with COVID-19. The Vanderbilt University Institutional Review Board determined that informed consent was not required, and all data were deidentified. The full data dictionary is provided in eTable 1 in Supplement 1. This study followed the Strengthening the Reporting of Observational Studies in Epidemiology (STROBE) reporting guideline.

### Statistical Analysis

We calculated bivariate frequencies to examine the associations among the baseline characteristics and receipt of convalescent plasma. The primary end point was death within 30 days of COVID-19 diagnosis. Living patients had their data censored at 30 days from diagnosis. Crude and adjusted hazard ratios (HRs) and 95% CIs to estimate the association between convalescent plasma use and 30-day all-cause mortality were calculated using Cox proportional hazards regression models. The primary analysis used propensity score matching to help account for the nonrandomized treatment administration of convalescent plasma.^14^ Individual propensities for receipt of convalescent plasma treatment were estimated using a multivariable probit regression model with baseline covariate adjustment using covariates that were determined a priori based on published literature and clinical importance: age, sex, race and ethnic groups, hematologic cancer type, cancer status, cancer treatment timing, ECOG performance status, obesity, presence of type 2 diabetes, hypertension, renal comorbidities, pulmonary comorbidities, receipt of cytotoxic chemotherapy within 3 months of COVID-19 diagnosis, and trimester of diagnosis (January to April 2020, May to August 2020, or September to December 2020). For matching, the nearest-neighbor method with a 1:1 ratio (treated units to control units) and 0.2 SD of the distance measure was applied to estimate the mean treatment effect.^15^ Marginal HRs along with 95% CIs based on cluster-robust SEs are reported. Kaplan-Meier survival curves were generated to compare survival probabilities using log-rank and stratified log-rank tests between convalescent plasma recipients and nonrecipients for unmatched and matched samples, respectively. We conducted several sensitivity analyses to explore the robustness of the findings for the primary hypothesis against the model specifications, such as varying the caliper size by ±0.1 and changing the matching order from the default maximum distance first to random order with different seeds. Exploratory subgroup analyses were conducted to determine whether patients with more severe illness (intensive care unit admission and/or mechanical ventilatory support) had differential outcome by convalescent plasma exposure.

We interpreted findings based on the 95% CIs for the estimated measures of association. Reported *P* values are 2-sided, with α < .05 considered to be statistically significant. Statistical analyses were performed using *R* software, version 4.0.3 with packages MatchIt and Survival (R Foundation for Statistical Computing).

## Results

As of January 21, 2021, the CCC19 registry contained 8209 case reports with complete baseline information. A total of 1761 patients (21.5%) had a primary or secondary hematologic cancer, with lymphoid cancers being the most common. After eligibility criteria were applied (eFigure 1 in Supplement 1), 966 patients (mean [SD] age, 65 [15] years; 539 [55.8%] male) were available for evaluation, of whom 143 (14.8%) received convalescent plasma treatment and 823 were untreated control patients (eFigure 2 in Supplement 1). Key patient characteristics are noted in Table 1; additional characteristics, including type of blood cancer and stage at cancer diagnosis, are provided in eTable 2 in Supplement 1. In the unmatched sample, convalescent plasma recipients were slightly younger and more likely to be male. A lower proportion of convalescent plasma recipients had pulmonary comorbidities and ECOG performance status of 2 or higher compared with the unexposed group. Convalescent plasma recipients were also more likely to be treated with corticosteroids, tocilizumab, and/or remdesivir and less likely to be treated with hydroxychloroquine. Overall, 512 patients (53.0%) had received systemic anticancer treatment within 3 months of COVID-19 diagnosis, with targeted therapies (monoclonal antibodies, small molecule inhibitors, and/or immunomodulators) being the most commonly received treatments. A total of 115 (22.5%) of those treated received an anti-CD20 antibody–containing regimen. Overall, 489 of 845 patients (57.9%) with an absolute lymphocyte count available had lymphopenia (lymphocyte count, <1500/μL [to convert to ×10^9^/L, multiply by 0.001]) at presentation; this proportion increased to 91 (79.1%) in patients who had received anti-CD20 antibodies. Propensity score matching was successful, with good balance achieved between the exposed and nonexposed groups (eFigures 3-5 in Supplement 1). The matched nonexposed group of 143 patients had more patients with multiple myeloma (47 [32.9%] vs 31 [21.7%]), fewer patients with CLL (12 [8.4%] vs 27 [18.9%]), and lower rates of disseminated disease at cancer diagnosis (100 [69.9%] vs 114 [79.7%]). Convalescent plasma recipients were more likely to require aggressive care (with 76 [53.1%] requiring intensive care unit admission and 45 [31.5%] requiring mechanical ventilatory support). Bleeding, sepsis, pulmonary complications, and congestive heart failure were more frequent in convalescent plasma recipients, with bleeding complications occurring in 16 (11.2%) convalescent plasma recipients vs 6 (4.2%) in propensity score–matched control patients, sepsis complications in 58 (40.6%) convalescent plasma recipients vs 32 (22.4%) propensity score–matched control patients, respiratory failure in 99 (69.2%) convalescent plasma recipients vs 66 (46.2%), and congestive heart failure in 10 (7%) convalescent plasma recipients vs fewer than 5 (<3.5%; entries other than missing or unknown with fewer than 5 patients were masked per CCC19 policy). Rates of hepatic and kidney injury were similar in both groups (8 [5.6%] of convalescent plasma recipients vs 7 [4.9%] of propensity score–matched control patients had acute hepatic injury and 37 [25.9%] of convalescent plasma recipients vs 39 [27.5%] of propensity score–matched control patients had acute kidney injury) (Table 2). Rates of venous thrombosis (15 [10.5%] vs 12 [8.4%]), arterial thrombotic events (5 [3.5%] vs <5 [<3.5%]), and arrhythmias (5 [3.5%] vs <5 [<3.5%]) were low and comparable in the convalescent plasma recipients vs the propensity score–matched controls.

**Table 1.** Characteristics of Patients Receiving or Not Receiving CP Before and After Propensity Score Matching^a^

Characteristic	Unmatched patients		Propensity score–matched patients	
	CP (n = 143)	No CP (n = 823)	CP (n = 143)	No CP (n = 143)
Time between hospitalization and first CP, median (IQR), d^b^	4 (1-8)	NA	4 (1-8)	NA
Age group, y				
18-39	12 (8.4)	54 (6.6)	12 (8.4)	15 (10.5)
40-59	37 (25.9)	174 (21.1)	37 (25.9)	38 (26.6)
60-69	45 (31.5)	233 (28.3)	45 (31.5)	45 (31.5)
70-79	31 (21.7)	209 (25.4)	31 (21.7)	28 (19.6)
≥80	18 (12.6)	153 (18.6)	18 (12.6)	17 (11.9)
Sex				
Male	82 (57.3)	457 (55.5)	82 (57.3)	85 (59.4)
Female	61 (42.7)	366 (44.5)	61 (42.7)	58 (40.6)
Race and ethnic group				
Non-Hispanic				
White	81 (56.6)	413 (50.2)	81 (56.6)	73 (51.0)
Black	19 (13.3)	174 (21.1)	19 (13.3)	29 (20.3)
Hispanic	26 (18.2)	152 (18.5)	26 (18.2)	24 (16.8)
Other	16 (11.2)	70 (8.5)	16 (11.2)	13 (9.1)
Missing or unknown	1 (0.7)	14 (1.7)	1 (0.7)	4 (2.8)
Comorbidity				
Hypertension	80 (55.9)	485 (58.9)	80 (55.9)	75 (52.4)
Obesity	53 (37.1)	282 (34.3)	53 (37.1)	53 (37.1)
Diabetes	38 (26.6)	259 (31.5)	38 (26.6)	41 (28.7)
Pulmonary	19 (13.3)	191 (23.2)	19 (13.3)	19 (13.3)
Renal	32 (22.4)	182 (22.1)	32 (22.4)	31 (21.7)
ECOG performance status				
0	37 (25.9)	196 (23.8)	37 (25.9)	40 (28.0)
1	53 (37.1)	267 (32.4)	53 (37.1)	57 (39.9)
≥2	17 (11.9)	172 (20.9)	17 (11.9)	15 (10.5)
Unknown	36 (25.2)	188 (22.8)	36 (25.2)	31 (21.7)
Baseline COVID-19 severity				
Mild	25 (16.9)	147 (17.9)	25 (16.9)	29 (20.3)
Moderate	79 (55.6)	503 (61.1)	79 (55.6)	87 (60.8)
Severe	34 (23.9)	166 (20.2)	34 (23.9)	24 (16.8)
Missing or unknown	5 (3.5)	7 (0.9)	5 (3.5)	3 (2.1)
Level of care required				
Hospitalization^c^	142 (99.3)	823 (100)	142 (99.3)	143 (100)
ICU admission	76 (53.1)	262 (31.8)	76 (53.1)	41 (28.7)
Mechanical ventilatory support	45 (31.5)	182 (22.1)	45 (31.5)	29 (20.3)
Other medications received during COVID-19 illness				
Corticosteroid	79 (55.2)	229 (27.8)	79 (55.2)	44 (30.8)
Remdesivir	72 (50.3)	153 (18.6)	72 (50.3)	35 (24.5)
Hydroxychloroquine	34 (23.8)	272 (33.0)	34 (23.8)	42 (29.4)
Tocilizumab	19 (13.3)	54 (6.6)	19 (13.3)	8 (5.6)
Type of hematologic cancer^d^				
Lymphoid	123 (86.0)	642 (78.0)	123 (86.0)	130 (90.9)
Myeloid	21 (14.7)	185 (22.5)	21 (14.7)	12 (8.4)
Cancer status				
Remission	45 (31.5)	251 (30.5)	45 (31.5)	50 (35.0)
Stable or responding	59 (41.3)	339 (41.2)	59 (41.3)	54 (37.8)
Progressing	18 (12.6)	125 (15.2)	18 (12.6)	13 (9.1)
Unknown	21 (14.7)	108 (13.1)	21 (14.7)	26 (18.2)

Abbreviations: CP, convalescent plasma; ECOG, Eastern Cooperative Oncology Group; ICU, intensive care unit; IQR, interquartile range; NA, not applicable.

^a^ Data are presented as number (percentage) of patients unless otherwise indicated.

^b^ Timing information was not initially available and was collected from sites after analysis. Information was collected for 107 of 143 (74.8%) of cases. For these cases, median time from COVID-19 diagnosis to first CP administration was 6.5 days (IQR, 2-14 days). Median time from COVID-19 diagnosis to first hospitalization was 0 days (IQR, 0-3 days).

^c^ Hospitalization status could not be verified for 1 patient receiving convalescent plasma; given that this treatment is given nearly universally in the hospital setting, the patient was retained for analysis.

^d^ Percentages total to more than 100% because some patients had multiple hematologic cancers (synchronous or metachronous).

**Table 2.** Selected Complications in CP Recipients, Propensity Score–Matched Control Patients, and All Control Patients

Complication	No. (%) of patients		
	CP recipients (n = 143)	No CP	
		Propensity score–matched control patients (n = 143)	Unmatched control patients (n = 823)
Cardiovascular complications			
Venous thromboembolism	15 (10.5)	12 (8.4)	63 (7.7)
Myocardial infarction and/or cerebrovascular accident	5 (3.5)	<5 (<3.5)^a^	26 (3.2)
Congestive heart failure	10 (7)	<5 (<3.5)^a^	45 (5.5)
Arrhythmia complications	5 (3.5)	<5 (<3.5)^a^	27 (3.3)
Pulmonary complications			
Respiratory failure	99 (69.2)	66 (46.2)	398 (48.4)
Pneumonia and/or pneumonitis	78 (54.5)	61 (42.7)	299 (36.3)
Acute respiratory distress syndrome	38 (26.6)	12 (8.4)	114 (13.9)
Other complications			
Bleeding complications	16 (11.2)	6 (4.2)	47 (5.7)
Sepsis complications	58 (40.6)	32 (22.4)	187 (22.7)
Acute hepatic injury	8 (5.6)	7 (4.9)	41 (5)
Acute kidney injury	37 (25.9)	39 (27.3)	222 (27)

Abbreviation: CP, convalescent plasma.

^a^ Entries other than missing or unknown with fewer than 5 patients are masked per COVID-19 and Cancer Consortium policy.

With a median follow-up period of 30 days (interquartile range, 21-90 days), 223 (23.1%) deaths occurred within 30 days of COVID-19 diagnosis (Table 3). The crude mortality rate was significantly lower in convalescent plasma recipients (19 of 143 [13.3%]) compared with nonrecipients (204 of 823 [24.8%]). This difference was statistically significant after adjustment in the overall comparison (HR, 0.60; 95% CI, 0.37-0.97; *P* = .03) and the propensity score–matched comparison (HR, 0.52; 95% CI, 0.29-0.92; *P* = .03) (Table 3 and Figure). Multiple additional sensitivity analyses, including analyses that used different caliper sizes for matching and analyses with randomized matching orders, found similar results. Among the 338 patients admitted to the intensive care unit, the crude mortality rate was significantly lower in convalescent plasma recipients compared with nonrecipients in the overall comparison (adjusted HR, 0.30; 95% CI, 0.16-0.56) and the propensity score–matched comparison (HR, 0.40; 95% CI, 0.20-0.80). Among the 227 patients requiring mechanical ventilatory support, the crude mortality rate was significantly lower in convalescent plasma recipients compared with nonrecipients in the overall comparison (HR, 0.23; 95% CI, 0.10-0.50) and the propensity score–matched comparison (HR, 0.32; 95% CI, 0.14-0.72) (Table 3; eFigure 6 in Supplement 1).

**Table 3.** Association Between Convalescent Plasma Use and Death Within the Crude Analysis, Multivariable Analysis, and Propensity Score Analyses

Variable	HR (95% CI) for death within 30 days
**Overall population**	
No. of events/No. of patients at risk (%)	223/966 (23.1)
Convalescent plasma	19/143 (13.3)
No convalescent plasma	204/823 (24.8)
Crude analysis^a^	0.47 (0.30-0.76)
Multivariable analysis^b^	0.60 (0.37-0.97)
Propensity score matching^c^	0.52 (0.29-0.92)
**Subgroup requiring ICU admission**	
No. of events/No. of patients at risk (%)	135/338 (39.9)
Convalescent plasma	12/76 (15.8)
No convalescent plasma	123/262 (46.9)
Crude analysis^a^	0.26 (0.14-0.47)
Multivariable analysis^b^	0.30 (0.16-0.56)
Propensity score matching^c^	0.40 (0.20-0.80)
**Subgroup requiring mechanical ventilatory support**	
No. of events/No. of patients at risk (%)	105/227 (46.3)
Convalescent plasma	8/45 (17.8)
No convalescent plasma	97/182 (53.3)
Crude analysis^a^	0.24 (0.16-0.49)
Multivariable analysis^b^	0.23 (0.10-0.50)
Propensity score matching^c^	0.32 (0.14-0.72)

Abbreviations: ICU, intensive care unit; HR, hazard ratio.

^a^ The HRs from the bivariable model in all patients from the unmatched study cohort.

^b^ The HRs form the multivariable stratified Cox proportional hazards regression model, with stratification by trimester of diagnosis with additional covariate adjustment.

^c^ Marginal HRs from propensity score–matched sample, constructed using 1:1 nearest neighbor matching with calipers of width equal to 0.2 of the SD of the distance measure.

**Figure.**
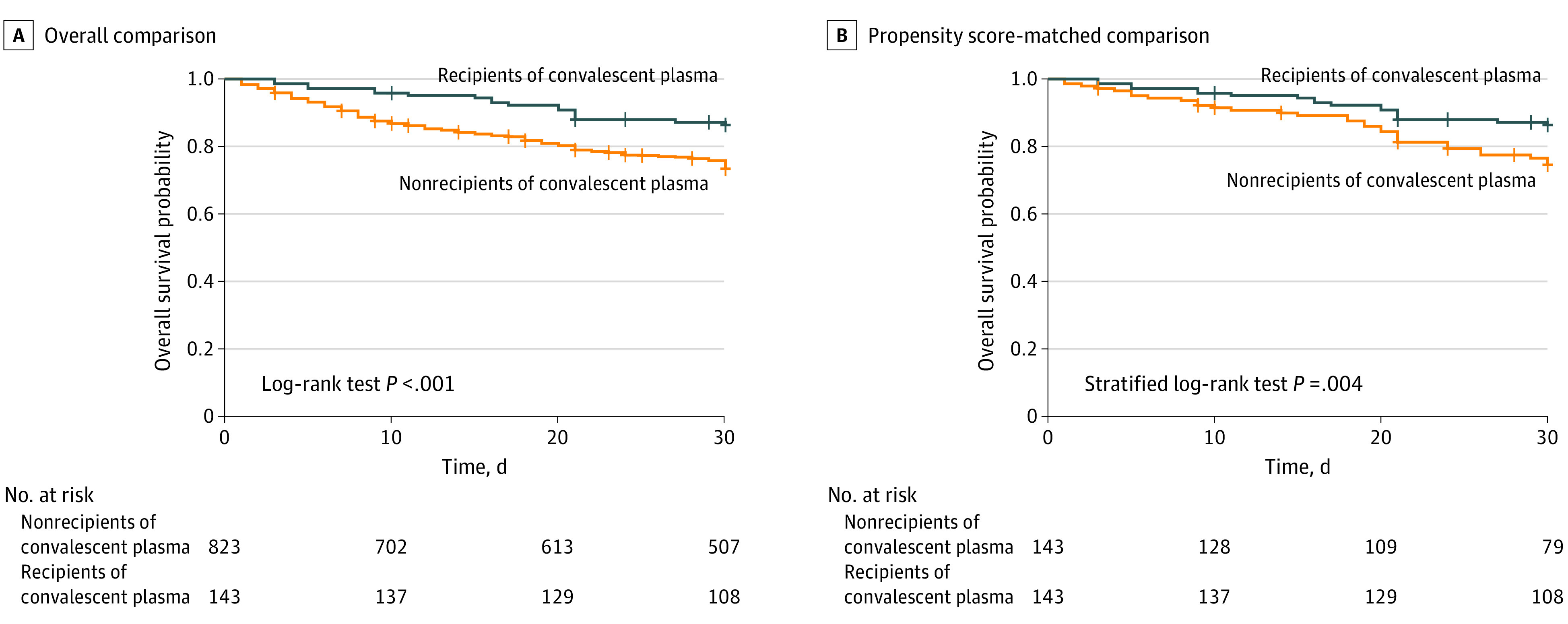
Overall Survival Rates Among Recipients vs Nonrecipients of Convalescent Plasma

## Discussion

This cohort study adds to the accumulating evidence supporting the efficacy of convalescent plasma treatment in patients with primary or secondary immunodeficiency, including those subjected to profound immunosuppression in the setting of hematopoietic stem cell transplantation.^16,17^ Patients with hematologic cancers may have immunodeficiencies from patient factors (including age), disease factors, and treatment factors. For example, in a single-center cohort of patients with CLL who had documented symptomatic COVID-19, 7 of 21 (33%) did not develop detectable anti–SARS-CoV-2 antibodies, notably lower than the 100% seroconversion rate observed in a noncancer population.^18,19^ A larger study^20^ recently found lower rates of seroconversion in patients with hematologic cancers, patients who received anti-CD20 antibodies, and hematopoietic transplant recipients. Several small studies^21,22,23^ have found improvement in clinical course after administration of convalescent plasma to patients with cancer, primarily hematologic cancers. Clinical improvement in COVID-19 symptoms within 48 hours of convalescent plasma transfusion was also reported in 16 of 17 patients with B-cell lymphopenia and prolonged COVID-19, 15 of whom had received anti-CD20 therapy in the 3 to 6 months before symptom onset.^23^

There is historical evidence of the efficacy of passive antibody therapy for infectious diseases when given early in the disease before the development of endogenous antibody responses, including in severe acute respiratory infections.^24,25,26^ On this basis, interventional trials of convalescent plasma treatment for patients with COVID-19 are ongoing; to our knowledge, only one of these, COVID19-Convalescent Plasma for Treating Patients With Active Symptomatic COVID 19 Infection (FALP-COVID),^27^ is specifically recruiting patients with cancer. Despite this notable absence of prospective clinical trials specifically for patients with cancer, there was widespread availability of convalescent plasma through the Expanded Access Program (EAP) and the subsequent US Food and Drug Administration Emergency Use Authorization (EUA). The EAP was open to more than 2800 acute care facilities in the US and territories.^28^ Presumably, most patients in this report received treatment through the EAP, EUA, or local non–cancer-specific clinical trials.

Lymphopenia was common in the study population, especially in patients with recent anti-CD20 treatment, as would be expected. We are unable to ascertain rates of hypogammaglobulinemia because this was not a routinely collected variable. The exact mechanism by which convalescent plasma may have mediated improved outcomes in the treated patients is likely multifactorial and could include reduction in viral load via enhanced clearance,^23^ reduction in secondary bacterial and fungal infections, neutralization of inflammatory cytokines that may otherwise promote a hyperinflammatory immune phenotype,^29^ and temporizing until the native immune system generates additional humoral and cell-mediated responses in the recovery phase after myelosuppressive or lymphodepleting anticancer therapy.

The current study is the largest such series reported to date, to our knowledge. Because of the multi-institutional nature of the data with more than 70 contributing institutions (eAppendix in Supplement 1), these findings are unlikely to be the result of specific practice patterns at certain institutions. Variables collected through this effort, such as cancer status, prior cancer treatments, and ECOG performance status, are not readily available through automated electronic health record extractions or claims databases. Notably, despite superior survival in the convalescent plasma group, there were considerably more sepsis and respiratory complications in this group. This finding likely reflects a higher severity of SARS-CoV-2 infection rather than complications from the treatment, although this possibility cannot be entirely excluded. Adverse effects of protein-rich infusions can include thromboses, kidney injury, and volume overload.^30,31,32^ It is reassuring that the rates of thromboses are low in both recipients and nonrecipients and the rates of acute kidney injury are similar. Although low, the rate of congestive heart failure in the convalescent plasma recipients is higher than in the matched control patients, and this finding bears additional scrutiny in larger cohorts.

### Limitations

This study has limitations, including its retrospective nature and unmeasured variables, such as the exact timing of convalescent plasma administration with respect to the date of COVID-19 diagnosis, the antibody titers and levels in the plasma that was administered, and whether repeat dosing was used. Although timing information is valuable, the feasibility of creating and maintaining a large, primarily voluntary, registry effort has necessitated study design decisions that would minimize the data entry burden for respondents; temporality is particularly burdensome and is only collected for very limited events (eg, death). As with many pharmacoepidemiological studies, immortal time bias is possible for both the time to convalescent plasma exposure in the treatment group and time from COVID-19 diagnosis to hospitalization in both recipients and nonrecipients.^33^ The registry data also lack details on timing and sequence of other treatment exposures in relation to convalescent plasma administration. Despite propensity matching, it is possible that residual confounding remains, and results should be interpreted with caution. For example, even after propensity matching, the convalescent plasma recipients received more corticosteroids and remdesivir. Although these agents have not been found to have a clear survival benefit in cancer populations,^34^ it is possible that at least part of the observed protective effect of convalescent plasma could be attributable to concomitant medications, including fewer administrations of hydroxychloroquine. There are some notable differences in blood cancer type and stage between the recipients and matched control patients, all of which would be expected to lead to worse outcomes in the recipients, where in fact the opposite was observed. These differences include more patients with multiple myeloma in the matched control patients, who have an intermediate prognosis.^35,36,37^ Conversely, more convalescent plasma recipients had CLL, which has been associated with poor outcomes.^38^ Convalescent plasma nonrecipients may have received less aggressive care overall because of factors other than COVID-19 (eg, advanced states of cancer); this possibility is partially addressed through adjustment for cancer status. In addition, fewer patients in the convalescent plasma–therapy group had disseminated disease at cancer diagnosis. Differential access to convalescent plasma because of health care system or socioeconomic factors, similar to what we previously observed for the investigational drug remdesivir, cannot be excluded.^34^ Although multi-institutional diversity is a strength of our study, it is also likely that heterogeneity in how stressed or overloaded a hospital was when the patient with COVID-19 was treated, as well as differences in academic and community settings, could have added additional potential confounding. It is possible that the findings in the first 30 days would not persist into later periods, which would require a more extended follow-up. Therefore, as with any observational study, causality cannot be inferred from these findings, but rather these findings can be viewed as contributing to the accumulating evidence regarding survival benefit with convalescent plasma treatment in patients with COVID-19 illness. Prospective randomized trials evaluating convalescent plasma in patients with hematologic cancers with attention to administration timing and consideration of repeated dosing are recommended.

## Conclusions

This cohort study found that convalescent plasma therapy was associated with a survival benefit in patients with hematologic cancers and COVID-19. If this finding should hold up in prospective clinical trials, convalescent plasma would be, to our knowledge, the first COVID-19 intervention with a survival benefit in this high-risk population.

## Notes

Supplement 1. **eAppendix.** Alphabetical List of Investigators by Institution That Contributed 1+ Patients to the Analysis. **eTable 1.** Data Dictionary Used for This Analysis **eTable 2.** Additional Patient Characteristics **eFigure 1.** CONSORT Diagram **eFigure 2.** Cumulative Patient Accrual **eFigure 3.** Distribution of Propensity Scores **eFigure 4.** Density Graph of the Propensity Scores Before and After Matching **eFigure 5.** Covariate Balance **eFigure 6.** Exploratory Subgroup Analysis

Supplement 2. **Nonauthor Collaborators.** COVID-19 and Cancer Consortium Members.
