# Supplementary material for: Association of Convalescent Plasma Therapy With Survival in Patients With Hematologic Cancers and COVID-19
Source: JAMA Oncol. 2021 Jun 17;7(8):1167–75. doi: 10.1001/jamaoncol.2021.1799 (PMC8377563; doi:10.1001/jamaoncol.2021.1799)
Supplement: Supplement 1. — eAppendix. Alphabetical List of Investigators by Institution That Contributed 1+ Patients to the Analysis. eTable 1. Data Dictionary Used for This Analysis eTable 2. Additional Patient Characteristics eFigure 1. CONSORT Diagram eFigure 2. Cumulative Patient Accrual eFigure 3. Distribution of Propensity Scores eFigure 4. Density Graph of the Propensity Scores Before and After Matching eFigure 5. Covariate Balance eFigure 6. Exploratory Subgroup Analysis [file jamaoncol-e211799-s001.pdf]

Supplementary Online Content

Thompson MA, Henderson JP, Shah PK, et al; for the COVID-19 and Cancer Consortium. Association of convalescent plasma therapy with survival in patients with hematologic cancers and COVID-19. *JAMA Oncol.* Published online June 17, 2021. doi:10.1001/jamaoncol.2021.1799

eAppendix. Alphabetical List of Investigators by Institution That Contributed 1+ Patients to the Analysis

eTable 1. Data Dictionary Used for This Analysis

eTable 2. Additional Patient Characteristics

eFigure 1. CONSORT Diagram

eFigure 2. Cumulative Patient Accrual

eFigure 3. Distribution of Propensity Scores

eFigure 4. Density Graph of the Propensity Scores Before and After Matching

eFigure 5. Covariate Balance

eFigure 6. Exploratory Subgroup Analysis

This supplementary material has been provided by the authors to give readers additional information about their work.

© 2021 American Medical Association. All rights reserved.

eAppendix. Alphabetical List of Investigators by Institution That Contributed 1+ Patients to the Analysis

Bolded = site PI/co-PIs); site co-investigators are listed alphabetically by last name

Balazs Halmos, MD; **Amit Verma, MBBS**; Benjamin A. Gartrell, MD; Sanjay Goel, MBBS; Nitin Ohri, MD; R. Alejandro Sica, MD; Astha Thakkar, MD (Albert Einstein College of Medicine, Montefiore Medical Center, Bronx, NY, USA)

Keith Stockerl-Goldstein, MD; Omar Butt, MD, PhD; Jian L. Campian, MD, PhD; Mark A. Fiala, MSW; Ryan Monahan, MBA; Alice Y. Zhou, MD, PhD (Alvin J. Siteman Cancer Center at Washington University School of Medicine and Barnes-Jewish Hospital, St. Louis, MO, USA)

Michael A. Thompson, MD, PhD, FASCO; Pamela Bohachek, RN; Daniel Mundt, MD; Mitrianna Streckfuss, MPH; Eyob Tadesse, MD (Aurora Cancer Care, Advocate Aurora Health, Milwaukee, WI, USA)

Philip E. Lammers, MD, MSCI (Baptist Cancer Center, Memphis, TN, USA)

Sanjay G. Revankar, MD, FIDSA (The Barbara Ann Karmanos Cancer Institute at Wayne State University School of Medicine, Detroit, MI, USA)

Orestis A. Panagiotou, MD, PhD; Pamela C. Egan, MD; Dimitrios Farmakiotis, MD, FACP, FIDSA; Hina Khan, MD; Adam J. Olszewski, MD (Brown University and Lifespan Cancer Institute, Providence, RI, USA)

Arturo Loaiza-Bonilla, MD, MSEd, FACP (Cancer Treatment Centers of America, AZ/GA/IL/OK/PA, USA)

Salvatore A. Del Prete, MD; Anne H. Angevine, MD; Michael H. Bar, MD, FACP; Anthony P. Gulati, MD; K. M. Steve Lo, MD; Jamie Stratton, MD; Paul L. Weinstein, MD (Carl & Dorothy Bennett Cancer Center at Stamford Hospital, Stamford, CT, USA)

Paolo Caimi, MD; Jill S. Barnholtz-Sloan, PhD; Jorge A. Garcia, MD, FACP; John M. Nakayama, MD (Case Comprehensive Cancer Center at Case Western Reserve University/University Hospitals, Cleveland, OH, USA)

Shilpa Gupta, MD; Nathan A. Pennell, MD, PhD, FASCO; Manmeet S. Ahluwalia, MD, FACP; Scott J. Dawsey, MD; Christopher A. Lemmon, MD; Amanda Nizam, MD (Cleveland Clinic, Cleveland, OH, USA)

Claire Hoppenot, MD; Ang Li, MD, MS (Dan L Duncan Comprehensive Cancer Center at Baylor College of Medicine, Houston, TX, USA)

Toni K. Choueiri, MD; Ziad Bakouny, MD, MSc; Gabrielle Bouchard, BS; Fiona J. Busser, BA; Jean M. Connors, MD; Catherine Curran, BA; George D. Demetri, MD, FASCO; Antonio Giordano, MD, PhD; Kaitlin Kelleher, BA; Anju Nohria, MD; Andrew Schmidt, MD; Grace Shaw, BA; Eli Van Allen, MD; Pier Vitale Nuzzo, MD, PhD; Wenxin (Vincent) Xu, MD; Rebecca L. Zon, MD (Dana-Farber Cancer Institute, Boston, MA, USA)

Tian Zhang, MD, MHS; Susan Halabi, PhD, FASCO (Duke Cancer Institute at Duke University Medical Center, Durham, NC, USA)

Gary H. Lyman, MD, MPH, FASCO, FRCP; Jerome J. Graber MD, MPH; Petros Grivas, MD, PhD; Ali Raza Khaki, MD; Elizabeth T. Loggers, MD, PhD; Ryan C. Lynch, MD; Elizabeth S. Nakasone, MD, PhD; Michael T. Schweizer, MD; Lisa Tachiki, MD; Shaveta Vinayak, MD, MS; Michael J. Wagner, MD; Albert Yeh, MD (Fred Hutchinson Cancer Research Center/University of Washington/Seattle Cancer Care Alliance, Seattle, WA, USA)

Na Tosha N. Gatson, MD, PhD (Geisinger Health System, PA, USA)

© 2021 American Medical Association. All rights reserved.

Sharad Goyal, MD; Minh-Phuong Huynh-Le, MD, MAS (George Washington University, Washington, DC, USA)

Lori J. Rosenstein, MD (Gundersen Health System, WI, USA)

Peter Paul Yu, MD, FACP, FASCO; Jessica M. Clement, MD; Ahmad Daher, MD; Mark Dailey, MD; Rawad Elias, MD; Asha Jayaraj, MD; Emily Hsu, MD; Alvaro G. Menendez, MD; Joerg Rathmann, MD; Oscar Serrano, MD (Hartford HealthCare Cancer Institute, Hartford, CT, USA)

Clara Hwang, MD; Shirish M. Gadgeel, MD (Henry Ford Cancer Institute, Henry Ford Hospital, Detroit, MI, USA)

Jessica E. Hawley, MD; Dawn Hershman, MD, MS, FASCO; Melissa K. Accordino, MD, MS; Divaya Bhutani, MD; Gary K. Schwartz, MD (Herbert Irving Comprehensive Cancer Center at Columbia University, New York, NY, USA)

Daniel Y. Reuben, MD, MS; Sarah Mushtaq, MD (Hollings Cancer Center at the Medical University of South Carolina, Charleston, SC, USA)

Eric H. Bernicker, MD (Houston Methodist Cancer Center, Houston, TX, USA)

John Deeken, MD; Danielle Shafer, DO (Inova Schar Cancer Institute, Fairfax, VA, USA)

Mark A. Lewis, MD; **Terence D. Rhodes, MD, PhD**; David M. Gill, MD; Clarke A. Low; MD (Intermountain Health Care, Salt Lake City, UT, USA)

Sarah Nagle, MD; Shannon McWeeney, PhD; Eneida R. Nemecek, MD, MS, MBA (Knight Cancer Institute at Oregon Health and Science University, Portland, OR, USA)

Howard A. Zaren, MD, FACS, Stephanie J. Smith, RN, MSN, OCN (Lewis Cancer & Research Pavilion @ St. Joseph's/Candler, Savannah, GA, USA)

Gayathri Nagaraj, MD; Mojtaba Akhtari, MD; Eric Lau, DO; Mark E. Reeves, MD (Loma Linda University Cancer Center, Loma Linda, CA, USA)

Stephanie Berg, DO; Destry Elms, MD (Loyola University Medical Center, Maywood, IL, USA)

Alicia K. Morgans, MD, MPH; Firas H. Wehbe, MD, PhD; Jessica Altman, MD; Michael Gurley, BA; Mary F. Mulcahy, MD (Lurie Cancer Center at Northwestern University, Chicago, IL, USA)

Eric B. Durbin, DrPH, MS (Markey Cancer Center at the University of Kentucky, Lexington, KY, USA)

Amit A. Kulkarni, MD; Heather H. Nelson, PhD, MPH; Surbhi Shah, MD (Masonic Cancer Center at the University of Minnesota, Minneapolis, MN, USA)

Rachel P. Rosovsky, MD, MPH; Kerry Reynolds, MD; Aditya Bardia, MD; Genevieve Boland, MD, PhD, FACS; Justin Gainor, MD; Leyre Zubiri, MD, PhD (Massachusetts General Hospital Cancer Center, Boston, MA, USA)

Thorvardur R. Halfdanarson, MD; Tanios Bekaii-Saab, MD; Aakash Desai, MD, MPH; Zhuoer Xie, MD, MS (Mayo Clinic, AZ/FL/MN, USA)

Ruben A. Mesa, MD, FACP; Mark Bonnen, MD; Daruka Mahadevan, MD, PhD; Amelie G. Ramirez, DrPH, MPH; Mary Salazar, ANP; Dimpy P. Shah, MD, PhD; Pankil K. Shah, MD, MSPH (Mays Cancer Center at UT Health San Antonio MD Anderson Cancer Center, San Antonio, TX, USA)

© 2021 American Medical Association. All rights reserved.

Gregory J. Riely, MD, PhD; **Elizabeth V. Robilotti MD, MPH**; Rimma Belenkaya, MA, MS; John Philip, MS (Memorial Sloan Kettering Cancer Center, New York, NY, USA)

Bryan Faller, MD (Missouri Baptist Medical Center, St. Louis, MO, USA)

Rana R. McKay, MD; Archana Ajmera, MSN, ANP-BC, AOCNP; Angelo Cabal, BS; Justin A. Shaya, MD (Moores Comprehensive Cancer Center at the University of California, San Diego, La Jolla, CA, USA)

Lisa B. Weissmann, MD, Chinmay Jani, MD (Mount Auburn Hospital, Cambridge, MA, USA)

Daniel G. Stover, MD; Daniel Addison, MD; James L. Chen, MD; Margaret E. Gatti-Mays, MD; Sachin R. Jhawar, MD; Vidhya Karivedu, MBBS; Maryam B. Lustberg, MD, MPH; Joshua D. Palmer, MD; Clement Pillainayagam, MD; Sarah Wall, MD; Nicole Williams, MD (The Ohio State University Comprehensive Cancer Center, Columbus, OH, USA)

Monika Joshi, MD, MRCP; Harry Menon, DO, MPH; Marc A. Rovito, MD, FACP (Penn State Health/Penn State Cancer Institute/St. Joseph Cancer Center, PA, USA)

Elizabeth A. Griffiths, MD; Amro Elshoury, MBBCh (Roswell Park Comprehensive Cancer Center, Buffalo, NY, USA)

Salma K. Jabbour, MD; Mansi R. Shah, MD (Rutgers Cancer Institute of New Jersey at Rutgers Biomedical and Health Sciences, New Brunswick, NJ, USA)

Babar Bashir, MD, MS; Christopher McNair, PhD; Sana Z. Mahmood, BA, BS; Vasil Mico, BS; Chaim Miller, BA; Andrea Verghese Rivera, MD (Sidney Kimmel Cancer Center at Thomas Jefferson University, Philadelphia, PA, USA)

Sumit A. Shah, MD, MPH; Elwyn C. Cabebe, MD; Michael J. Glover, MD; Alokkumar Jha, PhD; Lidia Schapira, MD, FASCO; Julie Tsu-Yu Wu, MD, PhD (Stanford Cancer Institute at Stanford University, Palo Alto, CA, USA)

Suki Subbiah, MD (Stanley S. Scott Cancer Center at LSU Health Sciences Center, New Orleans, LA, USA)

Daniel B. Flora, MD, PharmD; Goetz Kloecker, MD; Barbara B. Logan, MS (St. Elizabeth Healthcare, Edgewood, KY, USA)

Gilberto de Lima Lopes Jr., MD, MBA, FAMS, FASCO (Sylvester Comprehensive Cancer Center at the University of Miami Miller School of Medicine, Miami, FL, USA)

Karen Russell, MD, FACP; Brittany Stith, RN, BSN, OCN, CCRP (Tallahassee Memorial Healthcare, Tallahassee, FL, USA)

Natasha Edwin, MD; Melissa Smits, APC (ThedaCare Cancer Care, Appleton, WI, USA)

David Chism, MD; Susie Owenby, RN, CCRP (Thompson Cancer Survival Center, Knoxville, TN, USA)

Deborah B. Doroshow, MD, PhD; Matthew D. Galsky, MD; Huili Zhu, MD (Tisch Cancer Institute at the Icahn School of Medicine at Mount Sinai, New York, NY, USA)

Julie C. Fu, MD; Alyson Fazio, APRN-BC (Tufts Medical Center Cancer Center, Boston and Stoneham, MA, USA)

Jonathan Riess, MD, MS, Kanishka G. Patel, MD (UC Davis Comprehensive Cancer Center at the University of California at Davis, CA, USA)

© 2021 American Medical Association. All rights reserved.

Vadim S. Koshkin, MD; Daniel H. Kwon, MD (UCSF Helen Diller Family Comprehensive Cancer Center at the University of California at San Francisco, CA, USA)

Samuel M. Rubinstein, MD; **William A. Wood, MD, MPH**; Jessica Yasmine Islam, PhD, MPH; Vaibhav Kumar, MD (UNC Lineberger Comprehensive Cancer Center, Chapel Hill, NC, USA)

Trisha M. Wise-Draper, MD, PhD; Syed Ahmad, MD; Punita Grover, MD; Shuchi Gulati, MD; Jordan Kharofa, MD; Michelle Marcum, MS; Cathleen Park, MD (University of Cincinnati Cancer Center, Cincinnati, OH, USA)

Daniel W. Bowles, MD; Christoper L. Geiger, MD (University of Colorado Cancer Center, Aurora, CO, USA)

Merry-Jennifer Markham, MD, FACP, FASCO; Rohit Bishnoi, MD; Chintan Shah, MD (University of Florida Health Cancer Center, Gainesville, FL, USA)

Jared D. Acoba, MD; Young Soo Rho, MD, CM (University of Hawai'i Cancer Center, Honolulu, HI, USA)

Lawrence E. Feldman, MD; **Kent F. Hoskins, MD**; Gerald Gantt Jr., MD; Mahir Khan, MD; Ryan H. Nguyen, DO; Mary Pasquinelli, APN, DNP; Candice Schwartz, MD; Neeta K. Venepalli, MD, MBA (University of Illinois Hospital & Health Sciences System, Chicago, IL, USA)

Praveen Vikas, MD (University of Iowa Holden Comprehensive Cancer Center, Iowa City, IA, USA)

Elizabeth Wulff-Burchfield, MD; Anup Kasi MD, MPH (The University of Kansas Cancer Center, Kansas City, KS, USA)

Christopher R. Friese, PhD, RN, AOCN, FAAN; Leslie A. Fecher, MD (University of Michigan Rogel Cancer Center, Ann Arbor, MI, USA)

Blanche H. Mavromatis, MD; Ragneel Bijjula, MD; Qamar U. Zaman, MD (UPMC Western Maryland, Cumberland, MD, USA)

Jeremy L. Warner, MD, MS, FAMIA, FASCO; Alex Cheng, PhD; Elizabeth J. Davis, MD; Kyle T. Enriquez, MSc BS; Benjamin French, PhD; Erin A. Gillaspie, MD, MPH; Daniel Hausrath, MD; Cassandra Hennessy, MS; Chih-Yuan Hsu, PhD; Douglas B. Johnson, MD, MSCI; Xuanyi Li, BA; Sanjay Mishra, MS, PhD; Sonya A. Reid, MD, MPH; Brian I. Rini, MD, FACP, FASCO; Yu Shyr, PhD; David A. Slosky, MD; Carmen C. Solorzano, MD, FACS; Tianyi Sun, MS; Matthew D. Tucker, MD; Karen Vega-Luna; Lucy L. Wang, BA (Vanderbilt-Ingram Cancer Center at Vanderbilt University Medical Center, Nashville, TN, USA)

Matthew Puc, MD; Theresa M. Carducci, MSN, RN, CCRP; Karen J. Goldsmith, BSN, RN; Susan Van Loon, RN, CTR, CCRP (Virtua Health, Marlton, NJ, USA)

Robert L. Rice, MD, PhD (WellSpan Health, York, PA, USA)

Wilhelmina D. Cabalona, MD; Christine Pilar, BS, CCRC, ACRP-PM (Wentworth-Douglass Hospital, Dover, NH, USA)

Prakash Peddi, MD; **Lane R. Rosen, MD**; Briana Barrow McCollough, BSc, CCRC (Willis-Knighton Cancer Center, Shreveport, LA, USA)

Mehmet A. Bilen, MD; Deepak Ravindranathan, MD, MS (Winship Cancer Institute of Emory University, Atlanta, GA, USA)

Navid Hafez, MD, MPH; Roy Herbst, MD, PhD; Patricia LoRusso, DO, PhD; Tyler Masters, MS; Catherine Stratton, BA (Yale Cancer Center at Yale University School of Medicine, New Haven, CT, USA)

© 2021 American Medical Association. All rights reserved.

eTable 1. Data Dictionary used for this analysis.

The full CCC19 data dictionary and R code to generate the derived variables is publicly available on GitHub: https://github.com/covidncancer/CCC19_dictionary

Outcome description	Outcome variable name	Outcome values
30-day all-cause mortality (primary outcome measure)	der_dead30	0 = No; 1 = Yes; 99 = Unknown
Days from SARS-CoV-2 diagnosis to death	der_days_to_death_combined	Days (integer)
Median f/u time	der_median_fu	Days (integer)

Covariate description	Variable name	Possible covariate values
Receipt of convalescent plasma (primary stratification variable)	der_plasma	0 = No; 1 = Yes; 99 = Unknown
Age	der_age_cat	• 18-39 years<br>• 40-59 years<br>• 60-69 years<br>• 70-79 years<br>• 80+ years
Sex	der_sex	Male; Female
Race/ethnicity	der_race	• Non-Hispanic White; Hispanic; Non-Hispanic Black; Other
Smoking status	der_smoking2	• Never; Current or Former; Unknown
Obesity	der_obesity	0 = No; 1 = Yes; 99 = Unknown
Diabetes mellitus	der_dm2	0 = No; 1 = Yes; 99 = Unknown
Hypertension	der_htn	0 = No; 1 = Yes; 99 = Unknown
Renal comorbidities	der_renal	0 = No; 1 = Yes; 99 = Unknown
Pulmonary comorbidities	der_pulm	0 = No; 1 = Yes; 99 = Unknown
ECOG performance status	der_ecogcat2	0; 1; 2+; Unknown
Method of COVID-19 diagnosis	covid_19_diagnosis	1 = Suspected based on symptoms<br>11 = Suspected based on contact with confirmed case<br>2 = Suspected based on CXR findings<br>3 = Suspected based on CT scan findings<br>4 = Laboratory-confirmed<br>99 = Unknown
Baseline COVID-19 severity	severity_of_covid_19_v2	1 = Mild (no hospitalization required)<br>2 = Moderate (hospitalization indicated)<br>3 = Severe (ICU admission indicated)<br>99 = Unknown
Hospitalization (ever/never)	der_hosp	0 = No; 1 = Yes; 99 = Unknown
ICU admission (ever/never)	der_ICU	0 = No; 1 = Yes; 99 = Unknown
Mechanical ventilation (ever/never)	der_mv	0 = No; 1 = Yes; 99 = Unknown
Hydroxychloroquine given during COVID-19 illness	der_hcq	0 = No; 1 = Yes; 99 = Unknown

© 2021 American Medical Association. All rights reserved.

Remdesivir given during COVID-19 illness	der_rem	0 = No; 1 = Yes; 99 = Unknown
Corticosteroids given during COVID-19 illness	der_steroids_c19	0 = No; 1 = Yes; 99 = Unknown
Tocilizumab given during COVID-19 illness	der_toci	0 = No; 1 = Yes; 99 = Unknown
Absolute lymphocyte count	der_alc	• Normal<br>• High<br>• Low<br>• Not drawn/Not available<br>• Unknown
Lymphoid malignancy (primary and/or secondary)	der_Lymph	0 = No; 1 = Yes
Myeloid malignancy (primary and/or secondary)	der_Myeloid	0 = No; 1 = Yes
Chronic lymphocytic leukemia	der_CLL	0 = No; 1 = Yes
Plasma cell neoplasm	der_PCDs	0 = No; 1 = Yes
Cancer status	der_cancer_status	• Remission/NED<br>• Active, progressing<br>• Active, stable/responding<br>• Unknown
Timing of anti-cancer treatment	der_cancer_tx_timing	0 = More than 3 months prior to COVID-19<br>1 = Less than 2 weeks prior to COVID-19<br>2 = 2-4 weeks prior to COVID-19<br>3 = 1-3 months prior to COVID-19<br>88 = Never or starting after COVID-19 diagnosis<br>99 = Unknown
Cytotoxic chemotherapy within 3 months of COVID-19 diagnosis	der_any_cyto	0 = No; 1 = Yes; 99 = Unknown
Immunotherapy within 3 months of COVID-19 diagnosis	der_any_immuno	0 = No; 1 = Yes; 99 = Unknown
Targeted therapy within 3 months of COVID-19 diagnosis	der_any_targeted	0 = No; 1 = Yes; 99 = Unknown
Anti-CD20 antibody cancer treatment within 3 months of COVID-19	der_cd20	0 = No; 1 = Yes; 99 = Unknown
BTK inhibitor cancer treatment within 3 months of COVID-19	der_btki	0 = No; 1 = Yes; 99 = Unknown
Region of patient residence	der_region_v2	Non-US; Other; Undesignated US; US Midwest; US Northeast; US South; US West
Dummy variable corresponding to trimester of diagnosis, for case-control matching purposes	dummy_trimester_dx	x1, x2, x3 – the mapping of these variables to actual timed events is masked as required by the collaborator agreement.

© 2021 American Medical Association. All rights reserved.

eTable 2: Additional Patient Characteristics.

CP: COVID-19 convalescent plasma; NOS: not otherwise specified

Variable – no. (%)	Unmatched Patients		Propensity-Score Matched Patients	
	**CP (N = 143)**	**No CP (N = 823)**	**CP (N = 143)**	**No CP (N = 143)**
**Smoking**				
Never	82 (57.3)	446 (54.2)	82 (57.3)	71 (49.7)
Former/Current	58 (40.6)	349 (42.4)	58 (40.6)	68 (47.6)
Missing/Unknown	3 (2.1)	28 (3.4)	3 (2.1)	4 (2.8)
**Method of COVID-19 diagnosis**[a]				
Laboratory-confirmed	138 (96.5)	798 (97.0)	138 (96.5)	140 (97.9)
Suspected	5 (3.5)	23 (2.8)	5 (3.5)	<5 (<3.5)[i]
**Absolute lymphocyte count**				
Normal	33 (23.1)	240 (29.2)	33 (23.1)	46 (32.2)
Low[b]	79 (55.2)	410 (49.8)	79 (55.2)	72 (50.3)
High	13 (9.1)	70 (8.5)	13 (9.1)	8 (5.6)
Unknown/Not-drawn	18 (12.6)	103 (12.5)	18 (12.6)	17 (11.9)
**Type and subtype of hematologic malignancy**[c]				
Lymphoid	123 (86.0)	642 (78.0)	123 (86.0)	130 (90.9)
Multiple myeloma	31 (21.7)	161 (19.6)	31 (21.7)	47 (32.9)
Chronic lymphocytic leukemia	27 (18.9)	115 (14.0)	27 (18.9)	12 (8.4)
Non-Hodgkin lymphoma NOS	15 (10.5)	43 (5.2)	15 (10.5)	6 (4.2)
Acute lymphoblastic leukemia	14 (9.8)	43 (5.2)	14 (9.8)	9 (6.3)
Diffuse large B-cell lymphoma	14 (9.8)	100 (12.2)	14 (9.8)	17 (11.9)
Follicular lymphoma	8 (5.6)	39 (4.7)	8 (5.6)	10 (7.0)
Hodgkin lymphoma	5 (3.5)	45 (5.5)	5 (3.5)	10 (7.0)
Mantle cell lymphoma	5 (3.5)	13 (1.6)	5 (3.5)	<5 (<3.5)[i]
Other lymphoid[d]	5 (3.5)	35 (4.3)	5 (3.5)	9 (6.3)
T-cell and NK-cell neoplasm	<5 (<3.5)[i]	23 (2.8)	<5 (<3.5)[i]	<5 (<3.5)[i]
Marginal zone lymphoma	<5 (<3.5)[i]	22 (2.7)	<5 (<3.5)[i]	11 (7.7)
Plasma cell dyscrasia NOS	<5 (<3.5)[i]	15 (1.8)	<5 (<3.5)[i]	0 (0)
Lymphoproliferative disorder	<5 (<3.5)[i]	6 (0.7)	<5 (<3.5)[i]	0 (0)
Myeloid	21 (14.7)	185 (22.5)	21 (14.7)	12 (8.4)
Acute myeloid leukemia	9 (6.3)	72 (8.7)	9 (6.3)	7 (4.9)
Myelodysplastic syndrome	7 (4.9)	42 (5.1)	7 (4.9)	<5 (<3.5)[i]
Myeloproliferative neoplasm	<5 (<3.5)[i]	40 (4.9)	<5 (<3.5)[i]	0 (0)
Chronic myeloid leukemia	<5 (<3.5)[i]	33 (4.0)	<5 (<3.5)[i]	<5 (<3.5)[i]
Other myeloid	<5 (<3.5)[i]	6 (0.7)	<5 (<3.5)[i]	0 (0)

© 2021 American Medical Association. All rights reserved.

**Extent of disease at diagnosis[e]**				
Localized	20 (14.0)	112 (13.6)	20 (14.0)	25 (17.5)
Disseminated	114 (79.7)	625 (75.9)	114 (79.7)	100 (69.9)
Missing/Unknown	9 (6.3)	86 (10.5)	9 (6.3)	18 (12.6)
**Timing of anti-cancer treatment relative to COVID-19 diagnosis**				
<3 months	86 (60.1)	449 (54.6)	86 (60.1)	84 (58.7)
>3 months	39 (27.3)	237 (28.8)	39 (27.3)	40 (28.0)
Never or started after COVID-19 diagnosis	16 (11.2)	120 (14.6)	16 (11.2)	17 (11.9)
Unknown	2 (1.4)	17 (12.1)	2 (1.4)	2 (1.4)
**Type of anti-cancer treatment[f]**				
Targeted therapy[g]	63 (44.1)	316 (38.4)	63 (44.1)	63 (44.1)
Anti-CD20 antibodies[h]	23 (36.5)	92 (29.1)	23 (36.5)	17 (27.0)
BTK inhibitors[h]	10 (15.9)	30 (9.5)	10 (15.9)	6 (9.5)
Cytotoxic chemotherapy	25 (17.5)	213 (25.9)	25 (17.5)	29 (20.3)
Immunotherapy	14 (9.8)	18 (2.2)	14 (9.8)	<5 (<3.5)[i]
**U.S. census region of patient residence**				
Northeast	68 (47.6)	400 (48.6)	68 (47.6)	71 (49.7)
Midwest	35 (24.5)	183 (22.2)	35 (24.5)	26 (18.2)
West	22 (15.4)	88 (10.7)	22 (15.4)	11 (7.7)
South	18 (12.6)	152 (18.5)	18 (12.6)	35 (24.5)

[a]Percentages do not add to 100% because 2 patients had unknown method of diagnosis

[b]Defined as absolute lymphocyte count less than 1.5 x 10^9 cells/liter

[c]Percentages may add to more than 100% because some patients had multiple hematologic malignancies, including of the same subtype (synchronous or metachronous)

[d]Includes categories that cannot be further classified: aggressive lymphoma and indolent lymphoma

[e]Localized includes cancers that are anatomically staged I-III (e.g., lymphomas using the Ann Arbor staging system); disseminated includes cancers that are anatomically staged IV, and cancers that are by definition disseminated (i.e., multiple myeloma, acute and chronic leukemias, myelodysplastic syndrome, myeloproliferative neoplasms)

[f]Most recent treatment that was given within 3 months of COVID-19 diagnosis; patients may have received more than one modality

[g]Includes monoclonal antibodies, small molecule inhibitors, and immunomodulators

[h]These numbers may be underestimated; 70 of 512 (13.7%) patients on active treatment did not have any individual drug information provided to the registry

[i]Cells other than missing/unknown with fewer than 5 patients are masked per CCC19 policy

© 2021 American Medical Association. All rights reserved.

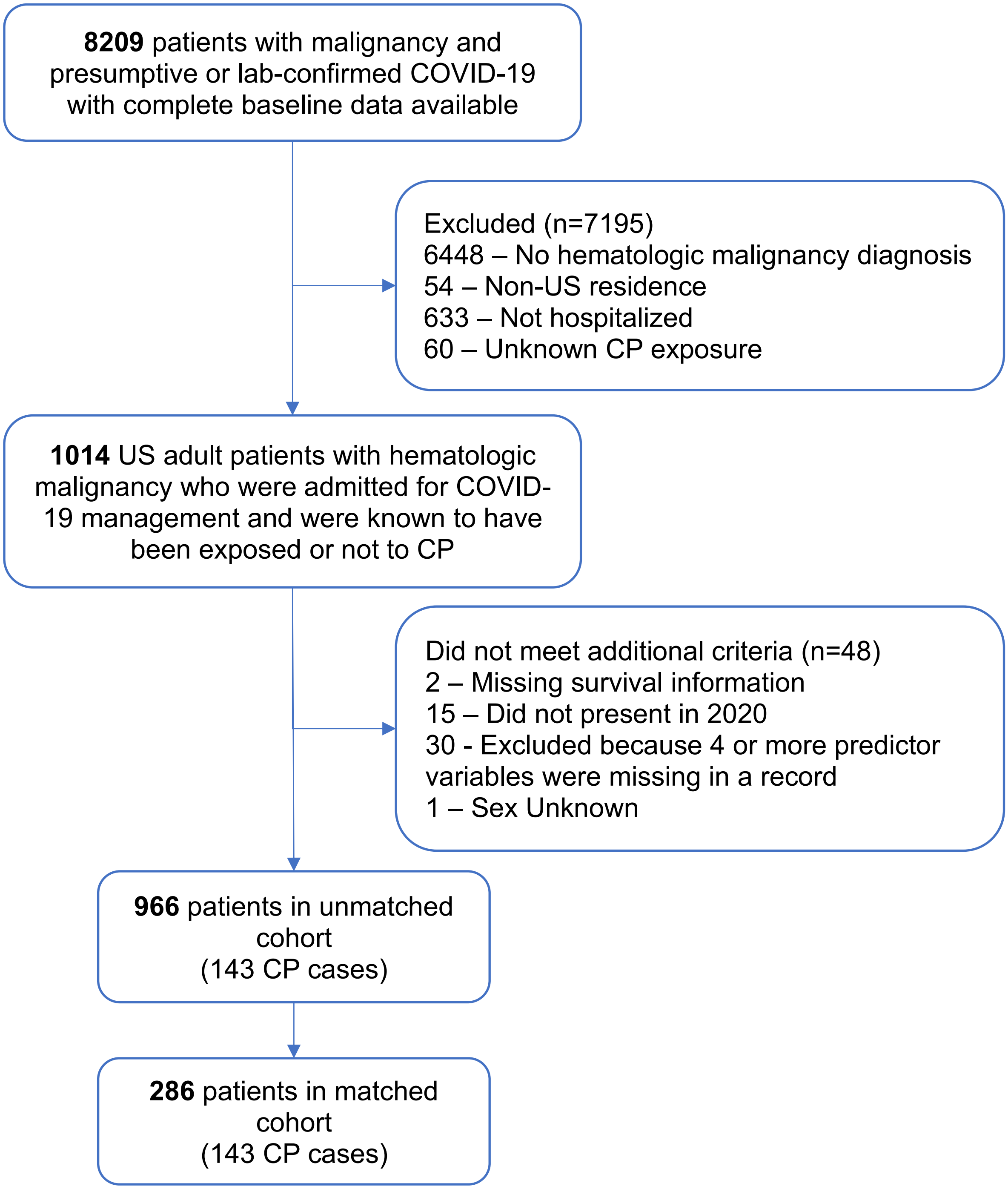

eFigure 1: CONSORT Diagram.

© 2021 American Medical Association. All rights reserved.

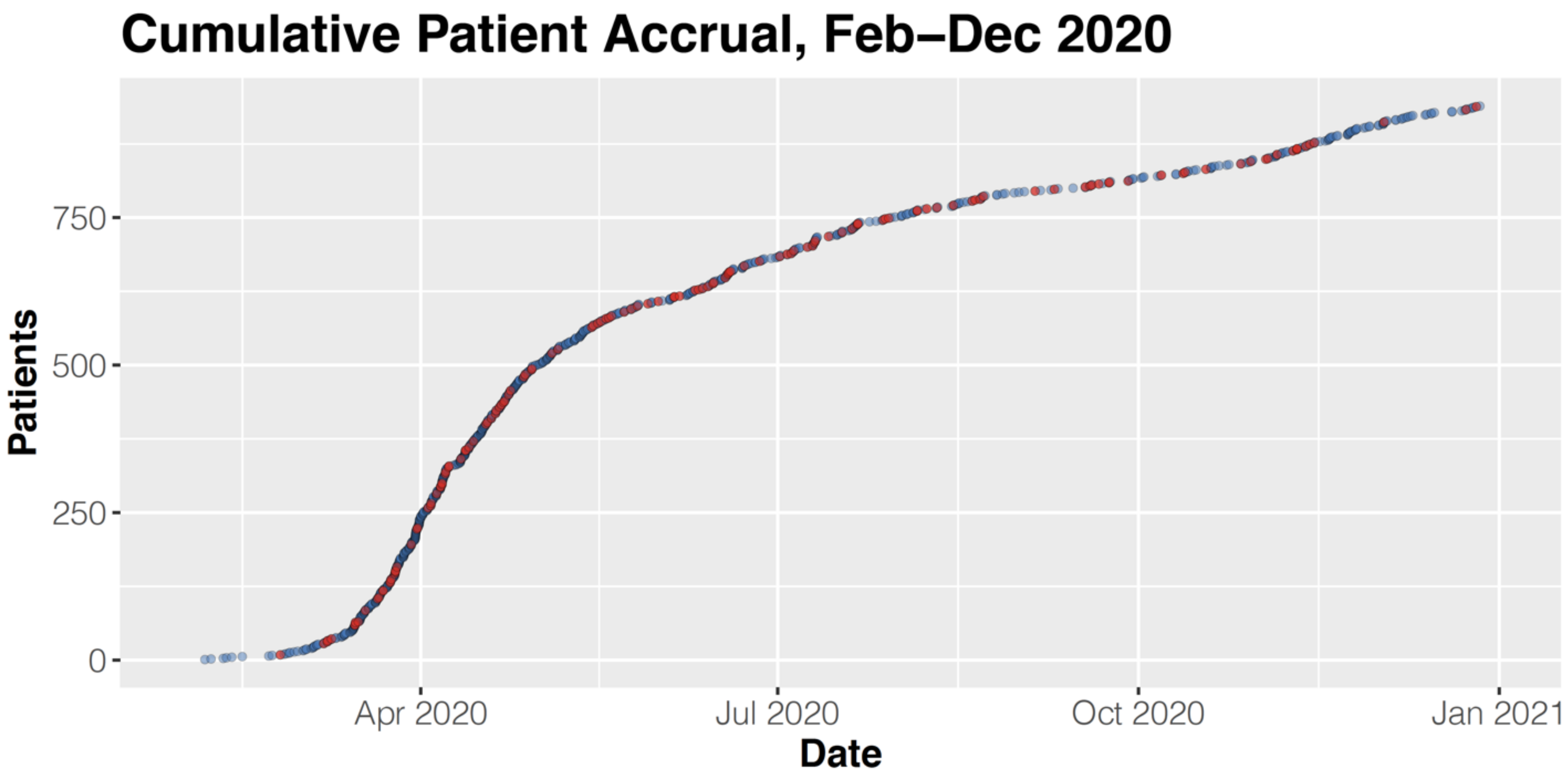

eFigure 2: Cumulative Patient Accrual. Red points represent patients who were treated with convalescent plasma; blue points represent patients who were not. The rate of accrual was highest in spring of 2020, with a continued near-linear growth in cases thereafter.

© 2021 American Medical Association. All rights reserved.

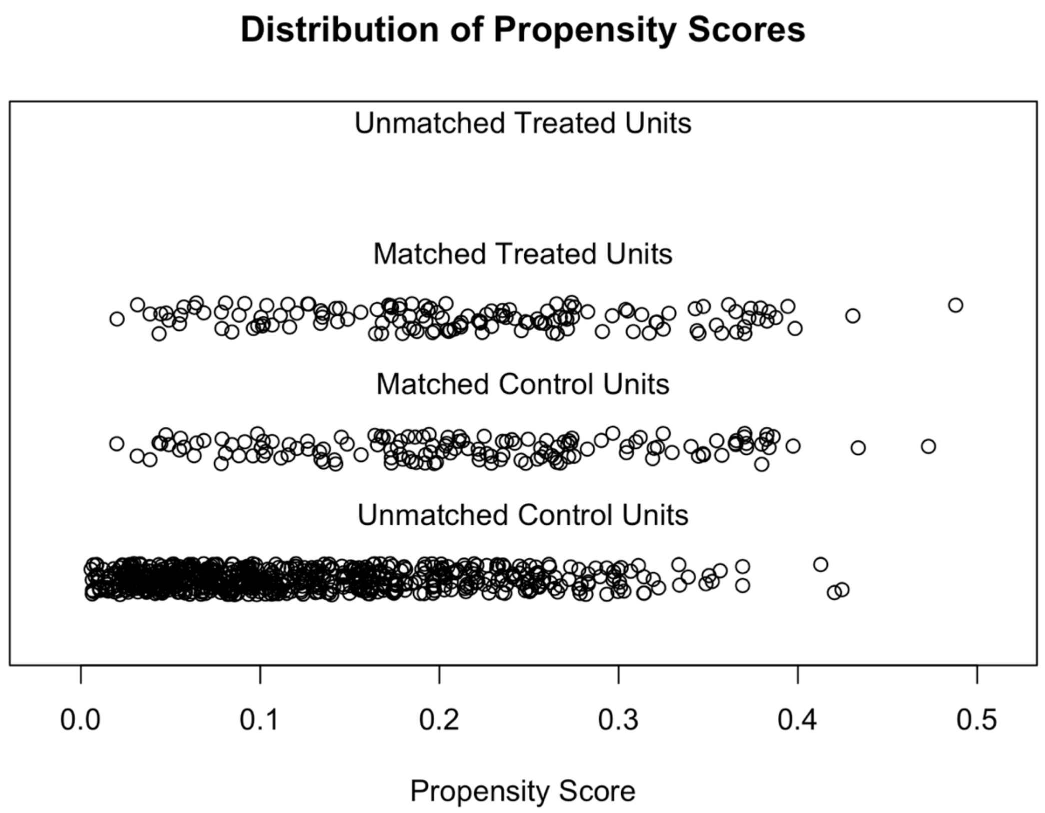

eFigure 3: Distribution of Propensity Scores

© 2021 American Medical Association. All rights reserved.

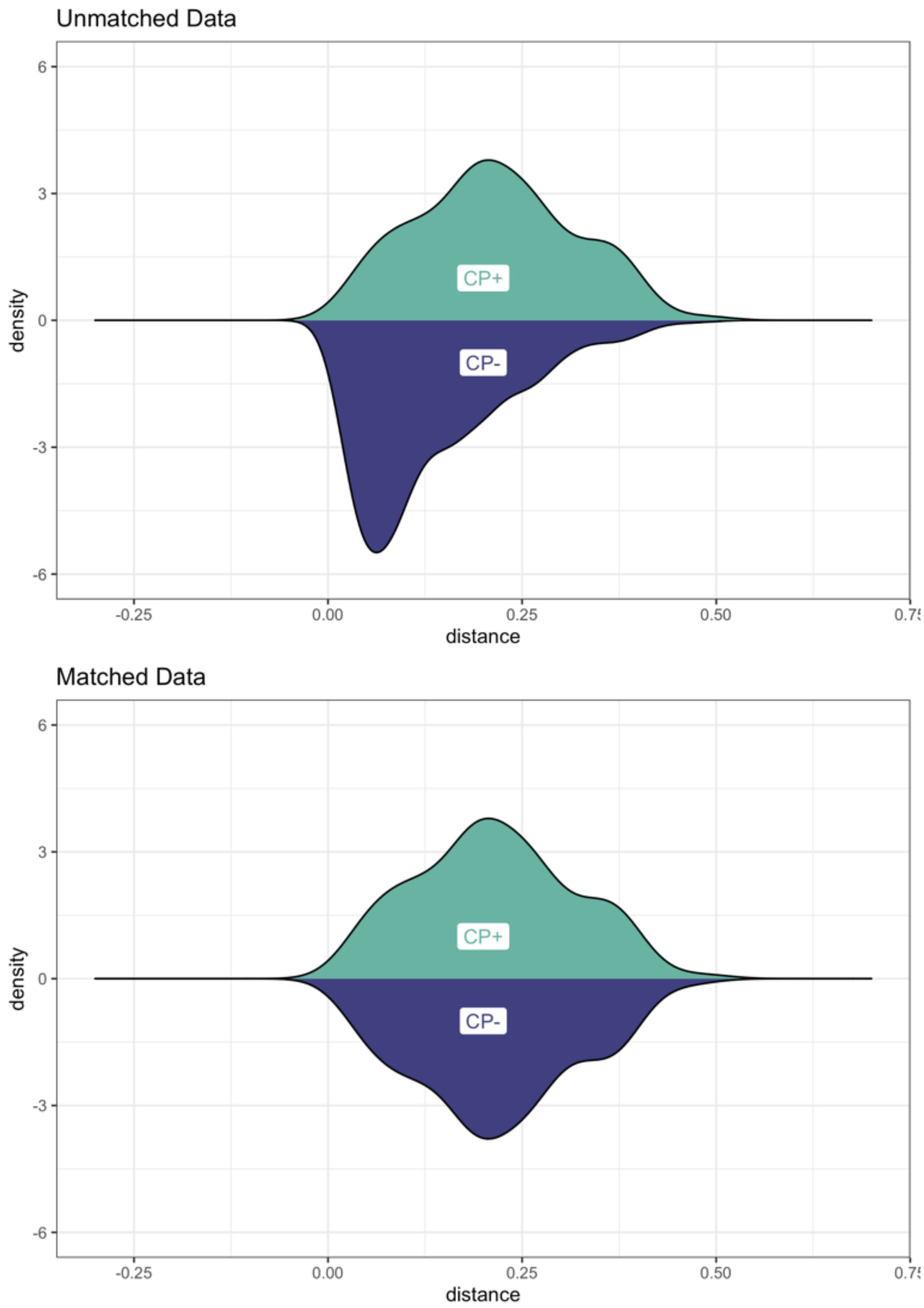

eFigure 4: Density Graph of the Propensity Scores Before and After Matching

© 2021 American Medical Association. All rights reserved.

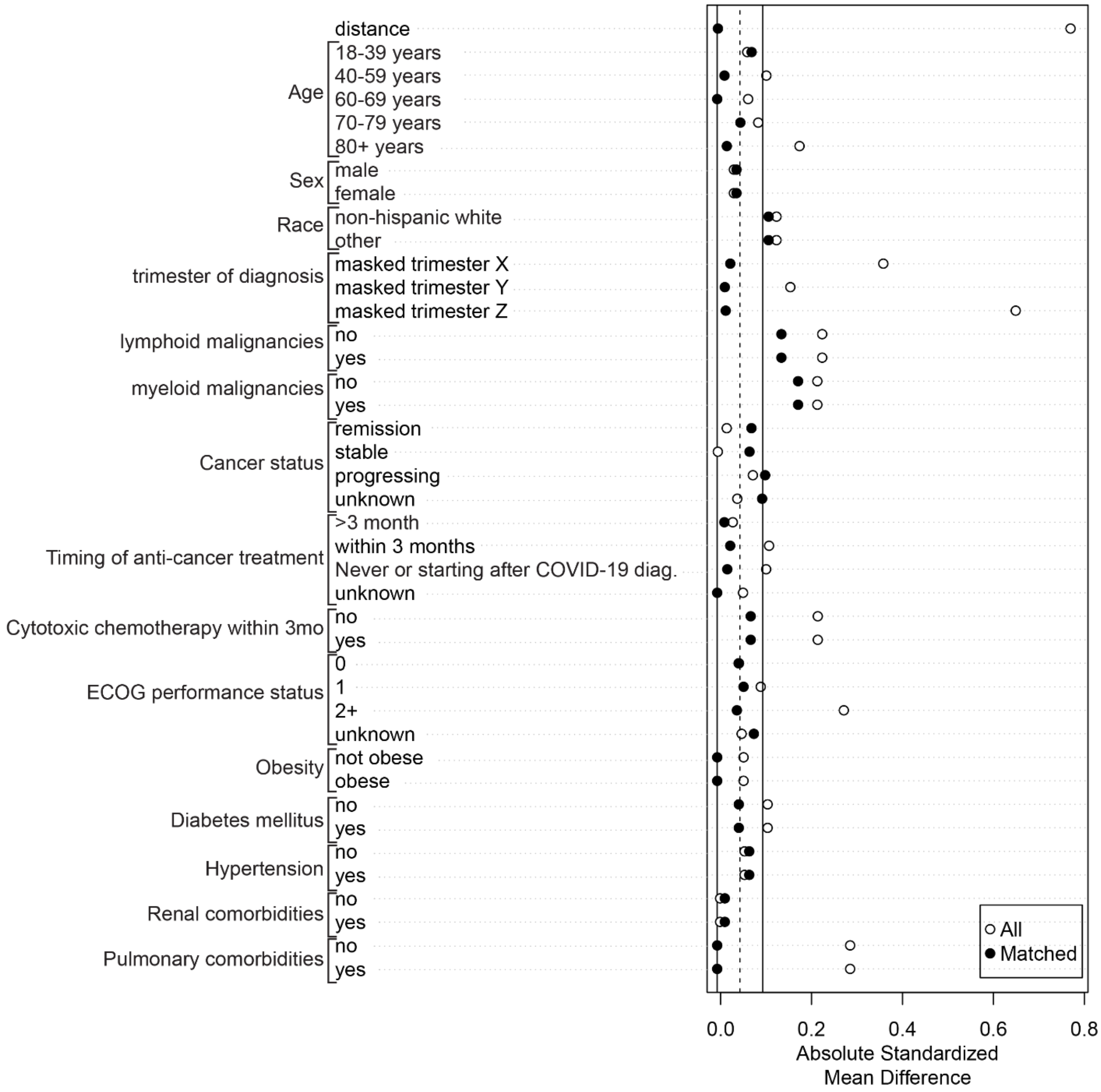

eFigure 5: Covariate Balance.

Distances closer to zero represent a closer match between the cases and controls; overall distance is shown at the top of the graphic.

© 2021 American Medical Association. All rights reserved.

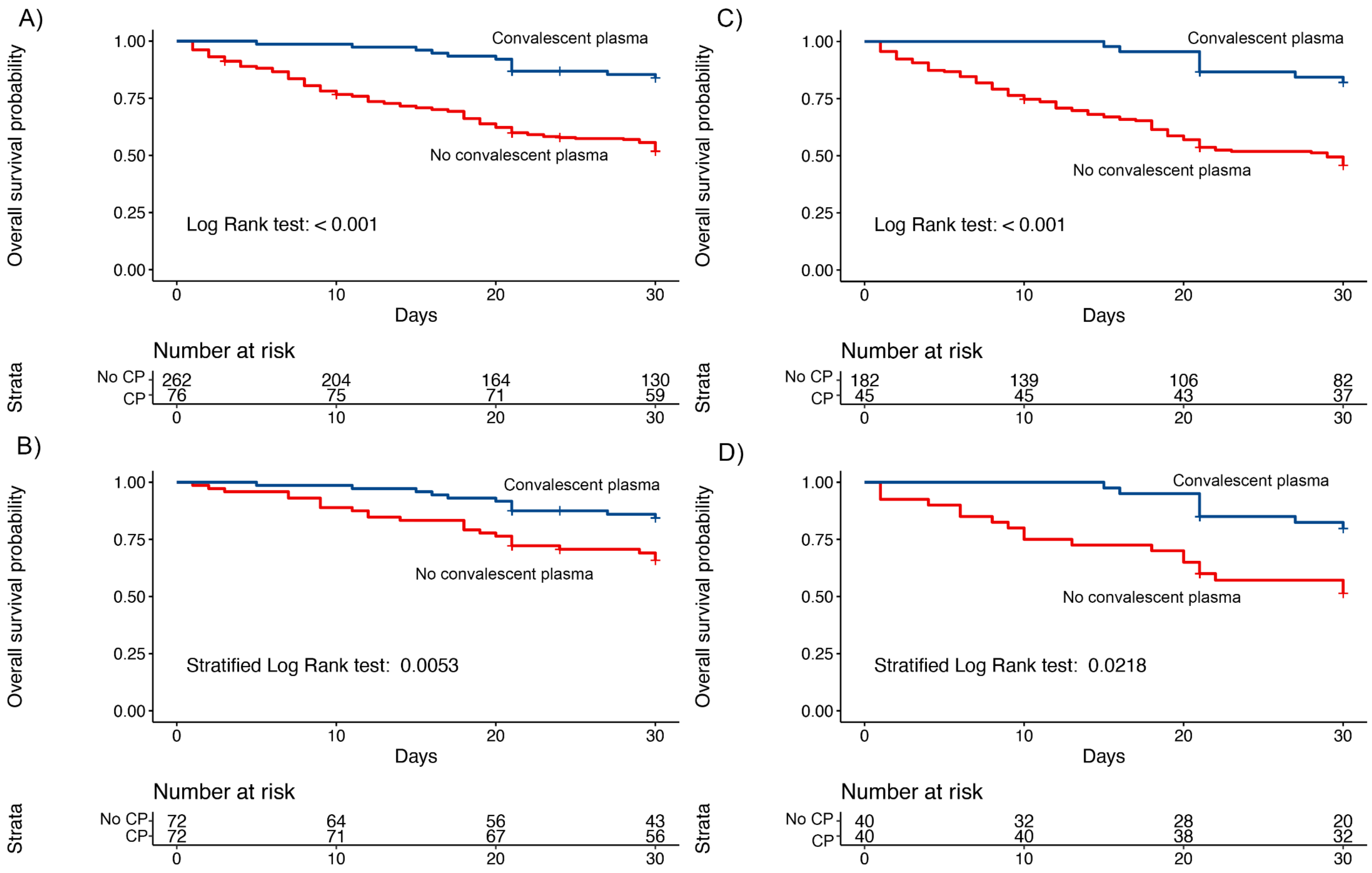

© 2021 American Medical Association. All rights reserved.

eFigure 6: Exploratory Subgroup Analyses. A-B) Patients admitted to the ICU: A) overall subpopulation and B) propensity-score matched population. C-D) Patients who required mechanical ventilation: C) overall subpopulation and D) propensity-score matched population.

© 2021 American Medical Association. All rights reserved.
