# Supplementary material for: Association of Convalescent Plasma Therapy With Survival in Patients With Hematologic Cancers and COVID-19
Source: JAMA Oncol. 2021 Jun 17;7(8):1167–75. doi: 10.1001/jamaoncol.2021.1799 (PMC8377563; doi:10.1001/jamaoncol.2021.1799)
Supplement: Supplement 2. — Nonauthor Collaborators. COVID-19 and Cancer Consortium Members. [file jamaoncol-e211799-s002.pdf]

Supplemental Online Content: Nonauthor Collaborators

*Indicates required information. Only first name, last name, and suffix will appear in PubMed.

*Group Name: The COVID-19 and Cancer Consortium							
***First Name and Middle Initial(s)**	***Last Name**	***Suffix (eg, Jr, III)**	Academic Degrees	Institution	Location (city, state/province, country)	Role or Contribution, eg, chair, principal investigator	Group (if more than 1 Group listed in the byline) and/or Subgroup (eg, Steering Committee)
Balazs	Halmos		MD	Albert Einstein College of Medicine, Montefiore Medical Center	Bronx, NY, USA	Site Co-PI	
Amit	Verma		MBBS	Albert Einstein College of Medicine, Montefiore Medical Center	Bronx, NY, USA	Site Co-PI	
Benjamin A	Gartrell		MD	Albert Einstein College of Medicine, Montefiore Medical Center	Bronx, NY, USA	Site Co-Investigator	
Sanjay	Goel		MBBS	Albert Einstein College of Medicine, Montefiore Medical Center	Bronx, NY, USA	Site Co-Investigator	
Nitin	Ohri		MD	Albert Einstein College of Medicine, Montefiore Medical Center	Bronx, NY, USA	Site Co-Investigator	
R Alejandro	Sica		MD	Albert Einstein College of Medicine, Montefiore Medical Center	Bronx, NY, USA	Site Co-Investigator	
Astha	Thakkar		MD	Albert Einstein College of Medicine, Montefiore Medical Center	Bronx, NY, USA	Site Co-Investigator	
Keith E	Stockerl-Goldstein		MD	Alvin J. Siteman Cancer Center at Washington University School of Medicine and Barnes-Jewish Hospital	St. Louis, MO, USA	Site PI	
Omar	Butt		MD, PhD	Alvin J. Siteman Cancer Center at Washington University School of Medicine and Barnes-Jewish Hospital	St. Louis, MO, USA	Site Co-Investigator	

Supplemental Online Content: Nonauthor Collaborators

*Indicates required information. Only first name, last name, and suffix will appear in PubMed.

***First Name and Middle Initial(s)**	***Last Name**	***Suffix (eg, Jr, III)**	Academic Degrees	Institution	Location (city, state/province, country)	Role or Contribution, eg, chair, principal investigator	Group (if more than 1 Group listed in the byline) and/or Subgroup (eg, Steering Committee)
Jian L	Campian		MD, PhD	Alvin J. Siteman Cancer Center at Washington University School of Medicine and Barnes-Jewish Hospital	St. Louis, MO, USA	Site Co-Investigator	
Mark A	Fiala		MSW	Alvin J. Siteman Cancer Center at Washington University School of Medicine and Barnes-Jewish Hospital	St. Louis, MO, USA	Site Co-Investigator	
Ryan	Monahan		MBA	Alvin J. Siteman Cancer Center at Washington University School of Medicine and Barnes-Jewish Hospital	St. Louis, MO, USA	Site Co-Investigator	
Alice Y	Zhou		MD, PhD	Alvin J. Siteman Cancer Center at Washington University School of Medicine and Barnes-Jewish Hospital	St. Louis, MO, USA	Site Co-Investigator	
Pamela	Bohachek		RN, CCRC	Aurora Cancer Care, Advocate Aurora Health	Milwaukee, WI, USA	Site Co-Investigator	
Daniel	Mundt		MD	Aurora Cancer Care, Advocate Aurora Health	Milwaukee, WI, USA	Site Co-Investigator	
Mitrianna	Streckfuss		MPH	Aurora Cancer Care, Advocate Aurora Health	Milwaukee, WI, USA	Site Co-Investigator	
Eyob	Tadesse		MD	Aurora Cancer Care, Advocate Aurora Health	Milwaukee, WI, USA	Site Co-Investigator	
Philip E	Lammers		MD, MSCI	Baptist Cancer Center	Memphis, TN, USA	Site PI	
Sanjay G	Revankar		MD, FIDSA	The Barbara Ann Karmanos Cancer Institute at Wayne State University School of Medicine	Detroit, MI, USA	Site PI	
Orestis A	Panagiotou		MD, PhD	Brown University and Lifespan Cancer Institute	Providence, RI, USA	Site PI	

Supplemental Online Content: Nonauthor Collaborators

*Indicates required information. Only first name, last name, and suffix will appear in PubMed.

***First Name and Middle Initial(s)**	***Last Name**	***Suffix (eg, Jr, III)**	Academic Degrees	Institution	Location (city, state/province, country)	Role or Contribution, eg, chair, principal investigator	Group (if more than 1 Group listed in the byline) and/or Subgroup (eg, Steering Committee)
Pamela C	Egan		MD	Brown University and Lifespan Cancer Institute	Providence, RI, USA	Site Co-Investigator	
Dimitrios	Farmakiotis		MD, FACP, FIDSA	Brown University and Lifespan Cancer Institute	Providence, RI, USA	Site Co-Investigator	
Hina	Khan		MD	Brown University and Lifespan Cancer Institute	Providence, RI, USA	Site Co-Investigator	
Adam J	Olszewski		MD	Brown University and Lifespan Cancer Institute	Providence, RI, USA	Site Co-Investigator	
Arturo	Loaiza-Bonilla		MD, MSEd, FACP	Cancer Treatment Centers of America	Philadelphia, PA, USA	Site PI	
Salvatore A	Del Prete		MD	Carl & Dorothy Bennett Cancer Center at Stamford Hospital	Stamford, CT, USA	Site PI	
Anne H	Angevine		MD	Carl & Dorothy Bennett Cancer Center at Stamford Hospital	Stamford, CT, USA	Site Co-Investigator	
Michael H	Bar		MD, FACP	Carl & Dorothy Bennett Cancer Center at Stamford Hospital	Stamford, CT, USA	Site Co-Investigator	
KM Steve	Lo		MD	Carl & Dorothy Bennett Cancer Center at Stamford Hospital	Stamford, CT, USA	Site Co-Investigator	
Jamie	Stratton		MD	Carl & Dorothy Bennett Cancer Center at Stamford Hospital	Stamford, CT, USA	Site Co-Investigator	
Paul L	Weinstein		MD	Carl & Dorothy Bennett Cancer Center at Stamford Hospital	Stamford, CT, USA	Site Co-Investigator	
Paolo F	Caimi		MD	Case Comprehensive Cancer Center at Case Western Reserve University/University Hospitals	Cleveland, OH, USA	Site PI	

Supplemental Online Content: Nonauthor Collaborators

*Indicates required information. Only first name, last name, and suffix will appear in PubMed.

*First Name and Middle Initial(s)	*Last Name	*Suffix (eg, Jr, III)	Academic Degrees	Institution	Location (city, state/province, country)	Role or Contribution, eg, chair, principal investigator	Group (if more than 1 Group listed in the byline) and/or Subgroup (eg, Steering Committee)
Jill S	Barnholtz-Sloan		PhD	Case Comprehensive Cancer Center at Case Western Reserve University/University Hospitals	Cleveland, OH, USA	Site Co-Investigator	
Jorge A	Garcia		MD, FACP	Case Comprehensive Cancer Center at Case Western Reserve University/University Hospitals	Cleveland, OH, USA	Site Co-Investigator	
John M	Nakayama		MD	Case Comprehensive Cancer Center at Case Western Reserve University/University Hospitals	Cleveland, OH, USA	Site Co-Investigator	
Shilpa	Gupta		MD	Cleveland Clinic	Cleveland, OH, USA	Site Co-PI	
Nathan A	Pennell		MD, PhD, FASCO	Cleveland Clinic	Cleveland, OH, USA	Site Co-PI	
Manmeet S	Ahluwalia		MD, FACP	Cleveland Clinic	Cleveland, OH, USA	Site Co-PI	
Scott J	Dawsey		MD	Cleveland Clinic	Cleveland, OH, USA	Site Co-Investigator	
Amanda	Nizam		MD	Cleveland Clinic	Cleveland, OH, USA	Site Co-Investigator	
Christopher A	Lemmon		MD	Cleveland Clinic	Cleveland, OH, USA	Site Co-Investigator	
Claire	Hoppenot		MD	Dan L Duncan Comprehensive Cancer Center at Baylor College of Medicine	Houston, TX, USA	Site Co-PI	
Ang	Li		MD, MS	Dan L Duncan Comprehensive Cancer Center at Baylor College of Medicine	Houston, TX, USA	Site Co-PI	

*Indicates required information. Only first name, last name, and suffix will appear in PubMed.

*First Name and Middle Initial(s)	*Last Name	*Suffix (eg, Jr, III)	Academic Degrees	Institution	Location (city, state/province, country)	Role or Contribution, eg, chair, principal investigator	Group (if more than 1 Group listed in the byline) and/or Subgroup (eg, Steering Committee)
Ziad	Bakouny		MD, MSc	Dana-Farber Cancer Institute	Boston, MA, USA	Site Co-Investigator	
Gabrielle	Bouchard		BS	Dana-Farber Cancer Institute	Boston, MA, USA	Site Co-Investigator	
Fiona J	Busser		BA	Dana-Farber Cancer Institute	Boston, MA, USA	Site Co-Investigator	
Jean M	Conners		MD	Dana-Farber Cancer Institute	Boston, MA, USA	Site Co-Investigator	
Catherine R	Curran		BA	Dana-Farber Cancer Institute	Boston, MA, USA	Site Co-Investigator	
George D	Demetri		MD, FASCO	Dana-Farber Cancer Institute	Boston, MA, USA	Site Co-Investigator	
Antonio	Giordano		MD, PhD	Dana-Farber Cancer Institute	Boston, MA, USA	Site Co-Investigator	
Kaitlin	Kelleher		BA	Dana-Farber Cancer Institute	Boston, MA, USA	Site Co-Investigator	
Anju	Nohria		MD	Dana-Farber Cancer Institute	Boston, MA, USA	Site Co-Investigator	
Andrew	Schmidt		MD	Dana-Farber Cancer Institute	Boston, MA, USA	Site Co-Investigator	
Grace	Shaw		BA	Dana-Farber Cancer Institute	Boston, MA, USA	Site Co-Investigator	
Eliezer M	Van Allen		MD	Dana-Farber Cancer Institute	Boston, MA, USA	Site Co-Investigator	
Pier Vitale	Nuzzo		MD, PhD	Dana-Farber Cancer Institute	Boston, MA, USA	Site Co-Investigator	
Wenxin	Xu		MD	Dana-Farber Cancer Institute	Boston, MA, USA	Site Co-Investigator	
Rebecca L	Zon		MD	Dana-Farber Cancer Institute	Boston, MA, USA	Site Co-Investigator	

*Indicates required information. Only first name, last name, and suffix will appear in PubMed.

*First Name and Middle Initial(s)	*Last Name	*Suffix (eg, Jr, III)	Academic Degrees	Institution	Location (city, state/province, country)	Role or Contribution, eg, chair, principal investigator	Group (if more than 1 Group listed in the byline) and/or Subgroup (eg, Steering Committee)
Tian	Zhang		MD, MHS	Duke Cancer Institute at Duke University Medical Center	Durham, NC, USA	Site PI	
Susan	Halabi		PhD, FASCO	Duke Cancer Institute at Duke University Medical Center	Durham, NC, USA	Site Co-Investigator	
Gary H	Lyman		MD, MPH, FASCO, FACP, FRCP	Fred Hutchinson Cancer Research Center/University of Washington/Seattle Cancer Care Alliance	Seattle, WA, USA	Site PI	
Jerome J	Graber		MD, MPH	Fred Hutchinson Cancer Research Center/University of Washington/Seattle Cancer Care Alliance	Seattle, WA, USA	Site Co-Investigator	
Petros	Grivas		MD, PhD	Fred Hutchinson Cancer Research Center/University of Washington/Seattle Cancer Care Alliance	Seattle, WA, USA	Site Co-Investigator	
Ali Raza	Khaki		MD	Fred Hutchinson Cancer Research Center/University of Washington/Seattle Cancer Care Alliance	Seattle, WA, USA	Site Co-Investigator	
Elizabeth T	Loggers		MD, PhD	Fred Hutchinson Cancer Research Center/University of Washington/Seattle Cancer Care Alliance	Seattle, WA, USA	Site Co-Investigator	
Ryan C	Lynch		MD	Fred Hutchinson Cancer Research Center/University of Washington/Seattle Cancer Care Alliance	Seattle, WA, USA	Site Co-Investigator	

*Indicates required information. Only first name, last name, and suffix will appear in PubMed.

*First Name and Middle Initial(s)	*Last Name	*Suffix (eg, Jr, III)	Academic Degrees	Institution	Location (city, state/province, country)	Role or Contribution, eg, chair, principal investigator	Group (if more than 1 Group listed in the byline) and/or Subgroup (eg, Steering Committee)
Elizabeth S	Nakasone		MD,PhD	Fred Hutchinson Cancer Research Center/University of Washington/Seattle Cancer Care Alliance	Seattle, WA, USA	Site Co-Investigator	
Michael T	Schweizer		MD	Fred Hutchinson Cancer Research Center/University of Washington/Seattle Cancer Care Alliance	Seattle, WA, USA	Site Co-Investigator	
Lisa ML	Tachiki		MD	Fred Hutchinson Cancer Research Center/University of Washington/Seattle Cancer Care Alliance	Seattle, WA, USA	Site Co-Investigator	
Shaveta	Vinayak		MD, MS	Fred Hutchinson Cancer Research Center/University of Washington/Seattle Cancer Care Alliance	Seattle, WA, USA	Site Co-Investigator	
Michael J	Wagner		MD	Fred Hutchinson Cancer Research Center/University of Washington/Seattle Cancer Care Alliance	Seattle, WA, USA	Site Co-Investigator	
Albert	Yeh		MD	Fred Hutchinson Cancer Research Center/University of Washington/Seattle Cancer Care Alliance	Seattle, WA, USA	Site Co-Investigator	
Na Tosha N	Gatson		MD, PhD	Geisinger Health System	PA, USA	Site PI	
Sharad	Goyal		MD	George Washington University	Washington, DC, USA	Site Co-PI	
Minh-Phuong	Huynh-Le		MD, MAS	George Washington University	Washington, DC, USA	Site Co-PI	
Lori J	Rosenstein		MD	Gundersen Health System	La Crosse, WI, USA	Site PI	
Jessica M	Clement		MD	Hartford HealthCare Cancer Institute	Hartford, CT, USA	Site Co-Investigator	

*Indicates required information. Only first name, last name, and suffix will appear in PubMed.

*First Name and Middle Initial(s)	*Last Name	*Suffix (eg, Jr, III)	Academic Degrees	Institution	Location (city, state/province, country)	Role or Contribution, eg, chair, principal investigator	Group (if more than 1 Group listed in the byline) and/or Subgroup (eg, Steering Committee)
Ahmad	Daher		MD	Hartford HealthCare Cancer Institute	Hartford, CT, USA	Site Co-Investigator	
Mark E	Dailey		MD	Hartford HealthCare Cancer Institute	Hartford, CT, USA	Site Co-Investigator	
Rawad	Elias		MD	Hartford HealthCare Cancer Institute	Hartford, CT, USA	Site Co-Investigator	
Emily	Hsu		MD	Hartford HealthCare Cancer Institute	Hartford, CT, USA	Site Co-Investigator	
Alvaro G	Menendez		MD	Hartford HealthCare Cancer Institute	Hartford, CT, USA	Site Co-Investigator	
Joerg	Rathmann		MD	Hartford HealthCare Cancer Institute	Hartford, CT, USA	Site Co-Investigator	
Oscar K	Serrano		MD, MBA, FACS	Hartford HealthCare Cancer Institute	Hartford, CT, USA	Site Co-Investigator	
Asha	Jayaraj		MD	Hartford HealthCare Cancer Institute	Hartford, CT, USA	Site Co-Investigator	
Shirish M	Gadgeel		MD	Henry Ford Cancer Institute, Henry Ford Hospital	Detroit, MI, USA	Site Co-Investigator	
Jessica E	Hawley		MD	Herbert Irving Comprehensive Cancer Center at Columbia University	New York, NY, USA	Site Co-PI	
Dawn L	Hershman		MD, MS, FASCO	Herbert Irving Comprehensive Cancer Center at Columbia University	New York, NY, USA	Site Co-PI	
Melissa K	Accordino		MD, MS	Herbert Irving Comprehensive Cancer Center at Columbia University	New York, NY, USA	Site Co-Investigator	
Divaya	Bhutani		MD	Herbert Irving Comprehensive Cancer Center at Columbia University	New York, NY, USA	Site Co-Investigator	

*Indicates required information. Only first name, last name, and suffix will appear in PubMed.

***First Name and Middle Initial(s)**	***Last Name**	***Suffix (eg, Jr, III)**	Academic Degrees	Institution	Location (city, state/province, country)	Role or Contribution, eg, chair, principal investigator	Group (if more than 1 Group listed in the byline) and/or Subgroup (eg, Steering Committee)
Gary K	Schwartz		MD	Herbert Irving Comprehensive Cancer Center at Columbia University	New York, NY, USA	Site Co-Investigator	
Daniel Y	Reuben		MD, MS	Hollings Cancer Center at the Medical University of South Carolina	Charleston, SC, USA	Site PI	
Sarah	Mushtaq		MD	Hollings Cancer Center at the Medical University of South Carolina	Charleston, SC, USA	Site Co-Investigator	
Eric H	Bernicker		MD	Houston Methodist Cancer Center	Houston, TX, USA	Site PI	
John	Deeken		MD	Inova Schar Cancer Institute	Fairfax, VA, USA	Site PI	
Danielle	Shafer		DO	Inova Schar Cancer Institute	Fairfax, VA, USA	Site Co-Investigator	
Mark A	Lewis		MD	Intermountain Health Care	Salt Lake City, UT, USA	Site Co-PI	
Terence D	Rhodes		MD, PhD	Intermountain Health Care	Salt Lake City, UT, USA	Site Co-PI	
David M	Gill		MD	Intermountain Health Care	Salt Lake City, UT, USA	Site Co-Investigator	
Clarke A	Low		MD	Intermountain Health Care	Salt Lake City, UT, USA	Site Co-Investigator	
Sarah	Nagle		MD	Knight Cancer Institute at Oregon Health and Science University	Portland, OR, USA	Site PI	
Brandon	Hayes-Lattin		MD, FACP	Knight Cancer Institute at Oregon Health and Science University	Portland, OR, USA	Site PI	
Shannon	McWeeney		PhD	Knight Cancer Institute at Oregon Health and Science University	Portland, OR, USA	Site Co-Investigator	

Supplemental Online Content: Nonauthor Collaborators

*Indicates required information. Only first name, last name, and suffix will appear in PubMed.

***First Name and Middle Initial(s)**	***Last Name**	***Suffix (eg, Jr, III)**	Academic Degrees	Institution	Location (city, state/province, country)	Role or Contribution, eg, chair, principal investigator	Group (if more than 1 Group listed in the byline) and/or Subgroup (eg, Steering Committee)
Eneida R	Nemecek		MD, MS, MBA	Knight Cancer Institute at Oregon Health and Science University	Portland, OR, USA	Site Co-Investigator	
Howard A	Zaren		MD, FACS	Lewis Cancer & Research Pavilion @ St Joseph's/Candler	Savannah, GA, USA	Site PI	
Stephanie J	Smith		RN, MSN, OCN	Lewis Cancer & Research Pavilion @ St Joseph's/Candler	Savannah, GA, USA	Site Co-Investigator	
Gayathri	Nagaraj		MD	Loma Linda University Cancer Center	Loma Linda, CA, USA	Site PI	
Mojtaba	Akhtari		MD	Loma Linda University Cancer Center	Loma Linda, CA, USA	Site Co-Investigator	
Eric	Lau		DO	Loma Linda University Cancer Center	Loma Linda, CA, USA	Site Co-Investigator	
Mark E	Reeves		MD, PhD	Loma Linda University Cancer Center	Loma Linda, CA, USA	Site Co-Investigator	
Stephanie	Berg		DO	Loyola University Medical Center	Maywood, IL, USA	Site PI	
Destry	Elms		MD	Loyola University Medical Center	Maywood, IL, USA	Site Co-Investigator	
Alicia K	Morgans		MD, MPH	Lurie Cancer Center at Northwestern University	Chicago, IL, USA	Site Co-PI	
Firas H	Wehbe		MD, PhD	Lurie Cancer Center at Northwestern University	Chicago, IL, USA	Site Co-PI	
Jessica	Altman		MD	Lurie Cancer Center at Northwestern University	Chicago, IL, USA	Site Co-Investigator	
Michael	Gurley		BA	Lurie Cancer Center at Northwestern University	Chicago, IL, USA	Site Co-Investigator	
Mary F	Mulcahy		MD	Lurie Cancer Center at Northwestern University	Chicago, IL, USA	Site Co-Investigator	

Supplemental Online Content: Nonauthor Collaborators

*Indicates required information. Only first name, last name, and suffix will appear in PubMed.

***First Name and Middle Initial(s)**	***Last Name**	***Suffix (eg, Jr, III)**	Academic Degrees	Institution	Location (city, state/province, country)	Role or Contribution, eg, chair, principal investigator	Group (if more than 1 Group listed in the byline) and/or Subgroup (eg, Steering Committee)
Eric B	Durbin		DrPH, MS	Markey Cancer Center at the University of Kentucky	Lexington, KY, USA	Site PI	
Amit A	Kulkarni		MD	Masonic Cancer Center at the University of Minnesota	Minneapolis, MN, USA	Site PI	
Heather H	Nelson		PhD, MPH	Masonic Cancer Center at the University of Minnesota	Minneapolis, MN, USA	Site Co-Investigator	
Surbhi	Shah		MD	Masonic Cancer Center at the University of Minnesota	Minneapolis, MN, USA	Site Co-Investigator	
Rachel P	Rosovsky		MD, MPH	Massachusetts General Hospital Cancer Center	Boston, MA, USA	Site Co-PI	
Kerry	Reynolds		MD	Massachusetts General Hospital Cancer Center	Boston, MA, USA	Site Co-PI	
Aditya	Bardia		MD	Massachusetts General Hospital Cancer Center	Boston, MA, USA	Site Co-Investigator	
Genevieve	Boland		MD, PhD, FACS	Massachusetts General Hospital Cancer Center	Boston, MA, USA	Site Co-Investigator	
Justin	Gainor		MD	Massachusetts General Hospital Cancer Center	Boston, MA, USA	Site Co-Investigator	
Leyre	Zubiri		MD, PhD	Massachusetts General Hospital Cancer Center	Boston, MA, USA	Site Co-Investigator	
Thorvardur R	Halfdanarson		MD	Mayo Clinic	Rochester, MN, USA	Site PI	
Tanios	Bekaii-Saab		MD, FACP	Mayo Clinic	Phoenix, AZ, USA	Site Co-Investigator	
Aakash	Desai		MD, MPH	Mayo Clinic	Rochester, MN, USA	Site Co-Investigator	
Ruben A	Mesa		MD, FACP	Mays Cancer Center at UT Health San Antonio MD Anderson Cancer Center	San Antonio, TX, USA	Site PI	

*Indicates required information. Only first name, last name, and suffix will appear in PubMed.

***First Name and Middle Initial(s)**	***Last Name**	***Suffix (eg, Jr, III)**	Academic Degrees	Institution	Location (city, state/province, country)	Role or Contribution, eg, chair, principal investigator	Group (if more than 1 Group listed in the byline) and/or Subgroup (eg, Steering Committee)
Mark	Bonnen		MD	Mays Cancer Center at UT Health San Antonio MD Anderson Cancer Center	San Antonio, TX, USA	Site Co-Investigator	
Daruka	Mahadevan		MD, PhD	Mays Cancer Center at UT Health San Antonio MD Anderson Cancer Center	San Antonio, TX, USA	Site Co-Investigator	
Amelie G	Ramirez		DrPH, MPH	Mays Cancer Center at UT Health San Antonio MD Anderson Cancer Center	San Antonio, TX, USA	Site Co-Investigator	
Mary	Salazar		DNP, MSN, RN, ANP-BC	Mays Cancer Center at UT Health San Antonio MD Anderson Cancer Center	San Antonio, TX, USA	Site Co-Investigator	
Rimma	Belenkaya		MA, MS	Memorial Sloan Kettering Cancer Center	New York, NY, USA	Site Co-Investigator	
John	Philip		MS	Memorial Sloan Kettering Cancer Center	New York, NY, USA	Site Co-Investigator	
Gregory J	Riely		MD, PhD	Memorial Sloan Kettering Cancer Center	New York, NY, USA	Site Co-PI	
Bryan	Faller		MD	Missouri Baptist Medical Center	St Louis, MO, USA	Site PI	
Rana R	McKay		MD	Moores Comprehensive Cancer Center at the University of California	San Diego, La Jolla, CA, USA	Site PI	
Archana	Ajmera		MSN, ANP-BC, AOCNP	Moores Comprehensive Cancer Center at the University of California	San Diego, La Jolla, CA, USA	Site Co-Investigator	
Angelo	Cabal		BS	Moores Comprehensive Cancer Center at the University of California	San Diego, La Jolla, CA, USA	Site Co-Investigator	

*Indicates required information. Only first name, last name, and suffix will appear in PubMed.

*First Name and Middle Initial(s)	*Last Name	*Suffix (eg, Jr, III)	Academic Degrees	Institution	Location (city, state/province, country)	Role or Contribution, eg, chair, principal investigator	Group (if more than 1 Group listed in the byline) and/or Subgroup (eg, Steering Committee)
Justin A	Shaya		MD	Moores Comprehensive Cancer Center at the University of California	San Diego, La Jolla, CA, USA	Site Co-Investigator	
Lisa B	Weissmann		MD	Mount Auburn Hospital	Cambridge, MA, USA	Site PI	
Chinmay	Jani		MD	Mount Auburn Hospital	Cambridge, MA, USA	Site Co-Investigator	
James L	Chen		MD	The Ohio State University Comprehensive Cancer Center	Columbus, OH, USA	Site Co-Investigator	
Margaret E	Gatti-Mays		MD	The Ohio State University Comprehensive Cancer Center	Columbus, OH, USA	Site Co-Investigator	
Sachin R	Jhawar		MD	The Ohio State University Comprehensive Cancer Center	Columbus, OH, USA	Site Co-Investigator	
Maryam B	Lustberg		MD, MPH	The Ohio State University Comprehensive Cancer Center	Columbus, OH, USA	Site Co-Investigator	
Joshua D	Palmer		MD	The Ohio State University Comprehensive Cancer Center	Columbus, OH, USA	Site Co-Investigator	
Clement	Pillainayagam		MD	The Ohio State University Comprehensive Cancer Center	Columbus, OH, USA	Site Co-Investigator	
Daniel G	Stover		MD	The Ohio State University Comprehensive Cancer Center	Columbus, OH, USA	Site PI	
Sarah	Wall		MD	The Ohio State University Comprehensive Cancer Center	Columbus, OH, USA	Site Co-Investigator	
Nicole O	Williams		MD	The Ohio State University Comprehensive Cancer Center	Columbus, OH, USA	Site Co-Investigator	
Vidhya	Karivedu		MBBS	The Ohio State University Comprehensive Cancer Center	Columbus, OH, USA	Site Co-Investigator	
Daniel	Addison		MD	The Ohio State University Comprehensive Cancer Center	Columbus, OH, USA	Site Co-Investigator	

Supplemental Online Content: Nonauthor Collaborators

*Indicates required information. Only first name, last name, and suffix will appear in PubMed.

***First Name and Middle Initial(s)**	***Last Name**	***Suffix (eg, Jr, III)**	Academic Degrees	Institution	Location (city, state/province, country)	Role or Contribution, eg, chair, principal investigator	Group (if more than 1 Group listed in the byline) and/or Subgroup (eg, Steering Committee)
Monika	Joshi		MD, MRCP	Penn State Health/Penn State Cancer Institute/St Joseph Cancer Center	PA, USA	Site Co-PI	
Harry	Menon		DO, MPH	Penn State Health/Penn State Cancer Institute/St Joseph Cancer Center	PA, USA	Site Co-PI	
Marc A	Rovito		MD, FACP	Penn State Health/Penn State Cancer Institute/St Joseph Cancer Center	PA, USA	Site Co-Investigator	
Amro	Elshoury		MBBCh	Roswell Park Comprehensive Cancer Center	Buffalo, NY, USA	Site Co-Investigator	
Salma K	Jabbour		MD	Rutgers Cancer Institute of New Jersey at Rutgers Biomedical and Health Sciences	New Brunswick, NJ, USA	Site PI	
Mansi R	Shah		MD	Rutgers Cancer Institute of New Jersey at Rutgers Biomedical and Health Sciences	New Brunswick, NJ, USA	Site Co-Investigator	
Babar	Bashir		MD, MS	Sidney Kimmel Cancer Center at Thomas Jefferson University	Philadelphia, PA, USA	Site PI	
Sana Z	Mahmood		BA, BS	Sidney Kimmel Cancer Center at Thomas Jefferson University	Philadelphia, PA, USA	Site Co-Investigator	
Christopher	McNair		PhD	Sidney Kimmel Cancer Center at Thomas Jefferson University	Philadelphia, PA, USA	Site Co-Investigator	
Vasil	Mico		BS	Sidney Kimmel Cancer Center at Thomas Jefferson University	Philadelphia, PA, USA	Site Co-Investigator	
Andrea Verghese	Rivera		MD	Sidney Kimmel Cancer Center at Thomas Jefferson University	Philadelphia, PA, USA	Site Co-Investigator	
Chaim	Miller		BA	Sidney Kimmel Cancer Center at Thomas Jefferson University	Philadelphia, PA, USA	Site Co-Investigator	

Supplemental Online Content: Nonauthor Collaborators

*Indicates required information. Only first name, last name, and suffix will appear in PubMed.

***First Name and Middle Initial(s)**	***Last Name**	***Suffix (eg, Jr, III)**	Academic Degrees	Institution	Location (city, state/province, country)	Role or Contribution, eg, chair, principal investigator	Group (if more than 1 Group listed in the byline) and/or Subgroup (eg, Steering Committee)
Elwyn	Cabebe		MD	Stanford Cancer Institute at Stanford University	Palo Alto, CA, USA	Site Co-Investigator	
Michael J	Glover		MD	Stanford Cancer Institute at Stanford University	Palo Alto, CA, USA	Site Co-Investigator	
Alokkumar	Jha		PhD	Stanford Cancer Institute at Stanford University	Palo Alto, CA, USA	Site Co-Investigator	
Lidia	Schapira		MD, FASCO	Stanford Cancer Institute at Stanford University	Palo Alto, CA, USA	Site Co-Investigator	
Sumit A	Shah		MD, MPH	Stanford Cancer Institute at Stanford University	Palo Alto, CA, USA	Site PI	
Julie Tsu-Yu	Wu		MD, PhD	Stanford Cancer Institute at Stanford University	Palo Alto, CA, USA	Site Co-Investigator	
Suki	Subbiah		MD	Stanley S Scott Cancer Center at LSU Health Sciences Center	New Orleans, LA, USA	Site PI	
Barbara B	Logan		MS	St Elizabeth Healthcare	Edgewood, KY, USA	Site Co-Investigator	
Goetz	Kloecker		MD	St Elizabeth Healthcare	Edgewood, KY, USA	Site Co-Investigator	
Gilberto de Lima	Lopes	Jr	MD, MBA, FAMS, FASCO	Sylvester Comprehensive Cancer Center at the University of Miami Miller School of Medicine	Miami, FL, USA	Site PI	
Karen	Russell		MD, FACP	Tallahassee Memorial Healthcare	Tallahassee, FL, USA	Site PI	
Brittany	Stith		BSN	Tallahassee Memorial Healthcare	Tallahassee, FL, USA	Site Co-Investigator	
Melissa	Smits		APC	ThedaCare Cancer Care	Appleton, WI, USA	Site Co-Investigator	
Natasha	Edwin		MD	ThedaCare Cancer Care	Appleton, WI, USA	Site PI	
David D	Chism		MD	Thompson Cancer Survival Center	Knoxville, TN, USA	Site PI	

*Indicates required information. Only first name, last name, and suffix will appear in PubMed.

***First Name and Middle Initial(s)**	***Last Name**	***Suffix (eg, Jr, III)**	Academic Degrees	Institution	Location (city, state/province, country)	Role or Contribution, eg, chair, principal investigator	Group (if more than 1 Group listed in the byline) and/or Subgroup (eg, Steering Committee)
Susie	Owenby		RN, CCRP	Thompson Cancer Survival Center	Knoxville, TN, USA	Site Co-Investigator	
Deborah B	Doroshow		MD, PhD	Tisch Cancer Institute at the Icahn School of Medicine at Mount Sinai	New York, NY, USA	Site PI	
Matthew D	Galsky		MD	Tisch Cancer Institute at the Icahn School of Medicine at Mount Sinai	New York, NY, USA	Site Co-Investigator	
Huili	Zhu		MD	Tisch Cancer Institute at the Icahn School of Medicine at Mount Sinai	New York, NY, USA	Site Co-Investigator	
Julie C	Fu		MD	Tufts Medical Center Cancer Center	Boston, MA, USA	Site PI	
Alyson	Fazio		APRN-BC	Tufts Medical Center Cancer Center	Boston, MA, USA	Site Co-Investigator	
Kanishka G	Patel		MD	UC Davis Comprehensive Cancer Center at the University of California at Davis	Davis, CA, USA	Site Co-Investigator	
Jonathan	Riess		MD, MS	UC Davis Comprehensive Cancer Center at the University of California at Davis	Davis, CA, USA	Site PI	
Daniel H	Kwon		MD	UCSF Helen Diller Family Comprehensive Cancer Center at the University of California at San Francisco	San Francisco, CA, USA	Site Co-Investigator	
Vaibhav	Kumar		MD	UNC Lineberger Comprehensive Cancer Center	Chapel Hill, NC, USA	Site Co-Investigator	
Jessica Y	Islam		PhD, MPH	UNC Lineberger Comprehensive Cancer Center	Chapel Hill, NC, USA	Site Co-Investigator	
William A	Wood		MD, MPH	UNC Lineberger Comprehensive Cancer Center	Chapel Hill, NC, USA	Site Co-PI	

*Indicates required information. Only first name, last name, and suffix will appear in PubMed.

***First Name and Middle Initial(s)**	***Last Name**	***Suffix (eg, Jr, III)**	Academic Degrees	Institution	Location (city, state/province, country)	Role or Contribution, eg, chair, principal investigator	Group (if more than 1 Group listed in the byline) and/or Subgroup (eg, Steering Committee)
Syed A	Ahmad		MD, FACS	University of Cincinnati Cancer Center	Cincinnati, OH, USA	Site Co-Investigator	
Punita	Grover		MD	University of Cincinnati Cancer Center	Cincinnati, OH, USA	Site Co-Investigator	
Shuchi	Gulati		MD	University of Cincinnati Cancer Center	Cincinnati, OH, USA	Site Co-Investigator	
Jordan	Kharofa		MD	University of Cincinnati Cancer Center	Cincinnati, OH, USA	Site Co-Investigator	
Michelle	Marcum		MS	University of Cincinnati Cancer Center	Cincinnati, OH, USA	Site Co-Investigator	
Cathleen	Park		MD	University of Cincinnati Cancer Center	Cincinnati, OH, USA	Site Co-Investigator	
Trisha M	Wise-Draper		MD, PhD	University of Cincinnati Cancer Center	Cincinnati, OH, USA	Site PI	
Daniel W	Bowles		MD	University of Colorado Cancer Center	Aurora, CO, USA	Site PI	
Christopher L	Geiger		MD	University of Colorado Cancer Center	Aurora, CO, USA	Site Co-Investigator	
Rohit	Bishnoi		MD	University of Florida Health Cancer Center	Gainesville, FL, USA	Site Co-Investigator	
Merry-Jennifer	Markham		MD, FACP, FASCO	University of Florida Health Cancer Center	Gainesville, FL, USA	Site PI	
Chintan	Shah		MD	University of Florida Health Cancer Center	Gainesville, FL, USA	Site Co-Investigator	
Jared D	Acoba		MD	University of Hawai'i Cancer Center	Honolulu, HI, USA	Site PI	
Young Soo	Rho		MD, CM	University of Hawai'i Cancer Center	Honolulu, HI, USA	Site Co-Investigator	
Lawrence E	Feldman		MD	University of Illinois Hospital & Health Sciences System	Chicago, IL, USA	Site Co-PI	

Supplemental Online Content: Nonauthor Collaborators

*Indicates required information. Only first name, last name, and suffix will appear in PubMed.

***First Name and Middle Initial(s)**	***Last Name**	***Suffix (eg, Jr, III)**	Academic Degrees	Institution	Location (city, state/province, country)	Role or Contribution, eg, chair, principal investigator	Group (if more than 1 Group listed in the byline) and/or Subgroup (eg, Steering Committee)
Kent F	Hoskins		MD	University of Illinois Hospital & Health Sciences System	Chicago, IL, USA	Site Co-PI	
Gerald	Gantt	Jr	MD	University of Illinois Hospital & Health Sciences System	Chicago, IL, USA	Site Co-Investigator	
Mahir	Khan		MD	University of Illinois Hospital & Health Sciences System	Chicago, IL, USA	Site Co-Investigator	
Ryan H	Nguyen		DO	University of Illinois Hospital & Health Sciences System	Chicago, IL, USA	Site Co-Investigator	
Mary M	Pasquinelli		APRN, FNP-BC	University of Illinois Hospital & Health Sciences System	Chicago, IL, USA	Site Co-Investigator	
Candice	Schwartz		MD	University of Illinois Hospital & Health Sciences System	Chicago, IL, USA	Site Co-Investigator	
Neeta K	Venepalli		MD, MBA	University of Illinois Hospital & Health Sciences System	Chicago, IL, USA	Site Co-Investigator	
Praveen	Vikas		MD	University of Iowa Holden Comprehensive Cancer Center	Iowa City, IA, USA	Site PI	
Anup	Kasi		MD, MPH	The University of Kansas Cancer Center	Kansas City, KS, USA	Site Co-Investigator	
Leslie A	Fecher		MD	University of Michigan Rogel Cancer Center	Ann Arbor, MI, USA	Site Co-PI	
Christopher R	Friese		PhD, RN, AOCN, FAAN	University of Michigan Rogel Cancer Center	Ann Arbor, MI, USA	Site Co-PI	
Blanche H	Mavromatis		MD	UPMC Western Maryland	Cumberland, MD, USA	Site PI	
Qamar U	Zaman		MD	UPMC Western Maryland	Cumberland, MD, USA	Site Co-Investigator	
Ragneel	Bijjula		MD	UPMC Western Maryland	Cumberland, MD, USA	Site Co-Investigator	

Supplemental Online Content: Nonauthor Collaborators

*Indicates required information. Only first name, last name, and suffix will appear in PubMed.

***First Name and Middle Initial(s)**	***Last Name**	***Suffix (eg, Jr, III)**	Academic Degrees	Institution	Location (city, state/province, country)	Role or Contribution, eg, chair, principal investigator	Group (if more than 1 Group listed in the byline) and/or Subgroup (eg, Steering Committee)
Alex	Cheng		PhD	Vanderbilt-Ingram Cancer Center at Vanderbilt University Medical Center	Nashville, TN, USA	Site Co-Investigator	
Elizabeth J	Davis		MD	Vanderbilt-Ingram Cancer Center at Vanderbilt University Medical Center	Nashville, TN, USA	Site Co-Investigator	
Benjamin	French		PhD	Vanderbilt-Ingram Cancer Center at Vanderbilt University Medical Center	Nashville, TN, USA	Site Co-Investigator	
Erin A	Gillaspie		MD, MPH	Vanderbilt-Ingram Cancer Center at Vanderbilt University Medical Center	Nashville, TN, USA	Site Co-Investigator	
Daniel J	Hausrath		MD	Vanderbilt-Ingram Cancer Center at Vanderbilt University Medical Center	Nashville, TN, USA	Site Co-Investigator	
Cassandra	Hennessy		MS	Vanderbilt-Ingram Cancer Center at Vanderbilt University Medical Center	Nashville, TN, USA	Site Co-Investigator	
Chih-Yuan	Hsu		PhD	Vanderbilt-Ingram Cancer Center at Vanderbilt University Medical Center	Nashville, TN, USA	Site Co-Investigator	
Douglas B	Johnson		MD, MSCI	Vanderbilt-Ingram Cancer Center at Vanderbilt University Medical Center	Nashville, TN, USA	Site Co-Investigator	
Xuanyi	Li		BA	Vanderbilt-Ingram Cancer Center at Vanderbilt University Medical Center	Nashville, TN, USA	Site Co-Investigator	
Sonya A	Reid		MD, MPH	Vanderbilt-Ingram Cancer Center at Vanderbilt University Medical Center	Nashville, TN, USA	Site Co-Investigator	

*Indicates required information. Only first name, last name, and suffix will appear in PubMed.

***First Name and Middle Initial(s)**	***Last Name**	***Suffix (eg, Jr, III)**	Academic Degrees	Institution	Location (city, state/province, country)	Role or Contribution, eg, chair, principal investigator	Group (if more than 1 Group listed in the byline) and/or Subgroup (eg, Steering Committee)
Brian I	Rini		MD, FACP, FASCO	Vanderbilt-Ingram Cancer Center at Vanderbilt University Medical Center	Nashville, TN, USA	Site Co-Investigator	
Yu	Shyr		PhD	Vanderbilt-Ingram Cancer Center at Vanderbilt University Medical Center	Nashville, TN, USA	Site Co-Investigator	
David A	Slosky		MD	Vanderbilt-Ingram Cancer Center at Vanderbilt University Medical Center	Nashville, TN, USA	Site Co-Investigator	
Carmen C	Solorzano		MD, FACS	Vanderbilt-Ingram Cancer Center at Vanderbilt University Medical Center	Nashville, TN, USA	Site Co-Investigator	
Matthew D	Tucker		MD	Vanderbilt-Ingram Cancer Center at Vanderbilt University Medical Center	Nashville, TN, USA	Site Co-Investigator	
Karen	Vega-Luna		MA	Vanderbilt-Ingram Cancer Center at Vanderbilt University Medical Center	Nashville, TN, USA	Site Co-Investigator	
Lucy L	Wang		BA	Vanderbilt-Ingram Cancer Center at Vanderbilt University Medical Center	Nashville, TN, USA	Site Co-Investigator	
Kyle T	Enriquez		MSc, BS	Vanderbilt-Ingram Cancer Center at Vanderbilt University Medical Center	Nashville, TN, USA	Site Co-Investigator	
Tianyi	Sun		MS	Vanderbilt-Ingram Cancer Center at Vanderbilt University Medical Center	Nashville, TN, USA	Site Co-Investigator	
Theresa M	Carducci		MSN, RN, CCRP	Virtua Health	Marlton, NJ, USA	Site Co-Investigator	
Matthew	Puc		MD	Virtua Health	Marlton, NJ, USA	Site PI	

Supplemental Online Content: Nonauthor Collaborators

*Indicates required information. Only first name, last name, and suffix will appear in PubMed.

***First Name and Middle Initial(s)**	***Last Name**	***Suffix (eg, Jr, III)**	Academic Degrees	Institution	Location (city, state/province, country)	Role or Contribution, eg, chair, principal investigator	Group (if more than 1 Group listed in the byline) and/or Subgroup (eg, Steering Committee)
Susan	Van Loon		RN, CTR, CCRP	Virtua Health	Marlton, NJ, USA	Site Co-Investigator	
Theresa M	Carducci		Carducci, MSN, RN, CCRP	Virtua Health	Marlton, NJ, USA	Site Co-Investigator	
Karen J	Goldsmith		BSN, RN	Virtua Health	Marlton, NJ, USA	Site Co-Investigator	
Robert L	Rice		MD, PhD	WellSpan Health	York, PA, USA	Site PI	
Wilhelmina D	Cabalona		MD	Wentworth-Douglass Hospital	Dover, NH, USA	Site PI	
Christine	Pilar		BS, CCRC, ACRP-PM	Wentworth-Douglass Hospital	Dover, NH, USA	Site Co-Investigator	
Briana	Barrow McCollough		BSc, CCRC	Willis-Knighton Cancer Center	Shreveport, LA, USA	Site Co-Investigator	
Prakash	Peddi		MD	Willis-Knighton Cancer Center	Shreveport, LA, USA	Site Co-PI	
Lane R	Rosen		MD	Willis-Knighton Cancer Center	Shreveport, LA, USA	Site Co-PI	
Mehmet A	Bilen		MD	Winship Cancer Institute of Emory University	Atlanta, GA, USA	Site PI	
Deepak	Ravindranathan		MD, MS	Winship Cancer Institute of Emory University	Atlanta, GA, USA	Site Co-Investigator	
Navid	Hafez		MD, MPH	Yale Cancer Center at Yale University School of Medicine	New Haven, CT, USA	Site PI	
Roy S	Herbst		MD, PhD	Yale Cancer Center at Yale University School of Medicine	New Haven, CT, USA	Site Co-Investigator	
Patricia	LoRusso		DO, PhD	Yale Cancer Center at Yale University School of Medicine	New Haven, CT, USA	Site Co-Investigator	
Tyler	Masters		MS	Yale Cancer Center at Yale University School of Medicine	New Haven, CT, USA	Site Co-Investigator	

Supplemental Online Content: Nonauthor Collaborators

*Indicates required information. Only first name, last name, and suffix will appear in PubMed.

***First Name and Middle Initial(s)**	***Last Name**	***Suffix (eg, Jr, III)**	Academic Degrees	Institution	Location (city, state/province, country)	Role or Contribution, eg, chair, principal investigator	Group (if more than 1 Group listed in the byline) and/or Subgroup (eg, Steering Committee)
Catherine	Stratton		BA, MPH	Yale Cancer Center at Yale University School of Medicine	New Haven, CT, USA	Site Co-Investigator	
